# UNC5B regulates epithelial-to-mesenchymal transition through a SRC-ZEB1 signaling axis to facilitate pancreatic cancer metastasis

**DOI:** 10.1038/s41388-026-03908-4

**Published:** 2026-08-01

**Authors:** Muhammad Sadeqi Nezhad, Chris R. Harris, Orjola Prela, Wade Narrow, Mitchell Breitenbach, Lan Wang, Shubhrika Jain, Jennifer L. Becker, Buket Bagci, Yansheng Hao, Aram F. Hezel, Scott A. Gerber, Stephano Mello, Tracy Withers, Darren R. Carpizo

**Affiliations:** 1 Clinical and Translational Science Institute, Translational Biomedical Science, University of Rochester School of Medicine and Dentistry, Rochester, NY USA; 2 Wilmot Cancer Institute, University of Rochester Medical Center, Rochester, NY USA; 3 Department of Surgery, Division of Surgical Oncology, University of Rochester School of Medicine and Dentistry, Rochester, NY USA; 4 Department of Pathology and Laboratory Medicine, University of Rochester School of Medicine and Dentistry, Rochester, NY USA; 5 Center for Advanced Research Technologies, Genomics Research Center, University of Rochester Medical Center, Rochester, NY USA; 6 Department of Microbiology and Immunology, University of Rochester School of Medicine and Dentistry, Rochester, NY USA; 7 Department of Biomedical Genetics, University of Rochester School of Medicine and Dentistry, Rochester, NY USA; 8 Lineberger Comprehensive Cancer Center, University of North Carolina, Chapel Hill, NC USA

**Keywords:** Metastasis, Cell signalling

## Abstract

Metastatic dissemination is the principal cause of death in pancreatic ductal adenocarcinoma (PDAC), yet the molecular determinants that enable this process remain poorly understood. Here, we identify the axon guidance receptor UNC5B as a central regulator of PDAC metastasis. Using both genetically engineered KPCU and orthotopic mouse models, we demonstrate that loss of UNC5B completely abolishes metastatic spread, reduces tumor proliferative capacity, increases intratumoral necrosis, confining tumors to the pancreas with no invasion into adjacent tissues or lymph nodes and preserving epithelial morphology. Mechanistically, UNC5B drives epithelial-to-mesenchymal transition (EMT) and invasion through activation of the SRC-ZEB1 axis. Notably, UNC5B specifically engages ZEB1 to drive EMT, without altering other canonical EMT transcription factors such as SNAIL or TWIST1. Pharmacological degradation of exogenous UNC5B using a targeted protein degrader (degron) modulated EMT and invasive behavior in PDAC cells. Acute depletion of UNC5B resulted in a marked reduction in EMT scores, accompanied by decreased ZEB1 and SRC levels. Together, these findings identify UNC5B as a central molecular hub governing metastatic competence in PDAC by promoting EMT and invasion.

**Figure:**
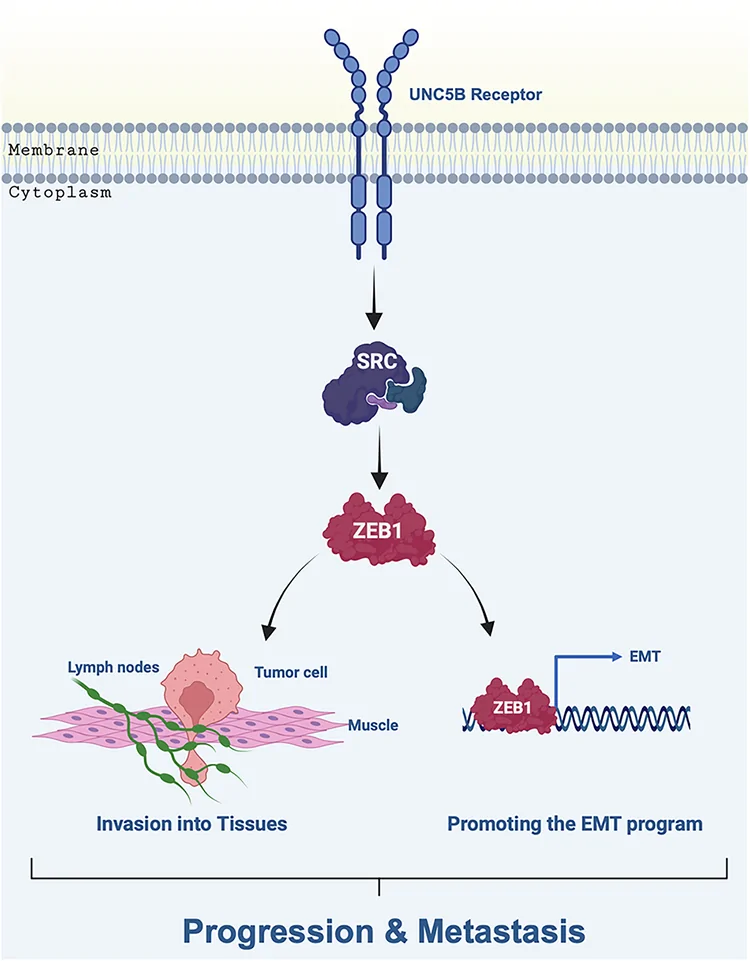

## Introduction

Pancreatic ductal adenocarcinoma (PDAC) is characterized by an exceptionally high propensity for distant metastasis, with most patients presenting with stage IV disease at diagnosis. Even among early-stage patients who undergo curative-intent surgery, the majority ultimately succumb to metastatic disease, indicating that disseminated tumor cells are present across all disease stages. Metastasis is therefore the predominant cause of mortality in PDAC, positioning the disease as the second-leading cause of cancer-related death in the United States by 2030 [1–3].

Metastatic progression is orchestrated by multiple cellular programs, including epithelial-mesenchymal transition (EMT), migration/invasion, anoikis resistance, intravasation, extravasation, and colonization of distant organs. EMT is a reversible program in which epithelial tumor cells lose polarity and adhesion, acquiring a mesenchymal phenotype that enhances motility and invasiveness [4]. Canonical EMT-inducing transcription factors (TFs) include SNAIL, SLUG, TWIST, and ZEB1 [5, 6]. In PDAC genetic ablation of Snail or Twist1 in the KPC (Pdx1-Cre; Kras^G12D/+^; Trp53^R172H/+^) mouse model did not impair tumor initiation, invasion, or metastasis, suggesting that Snail and Twist1 are largely dispensable for PDAC metastatic progression [7]. In contrast, deletion of Zeb1 markedly reduced invasion and metastatic burden in the same model, highlighting a critical and non-redundant role for EMT in driving PDAC metastasis [8]. The upstream molecular mechanism that drives Zeb-1 dependent EMT in PDAC up to now has been undefined.

We previously identified the axon guidance molecule Netrin-1 as upregulated in PDAC metastasis and inhibition of Netrin-1 using a monoclonal antibody inhibits metastasis and improves survival in murine models of PDAC liver metastasis [9]. Netrin-1 signals through the Dependence Receptor (DR) family which include Deleted in Colon Cancer (DCC) and Uncoordinated 5 family members. We found the dominant DR in PDAC is UNC5B [9]. These receptors have been named for their ability to impart both positive signals (proliferation, migration) upon ligand binding and negative signals (apoptosis) upon ligand deprivation [10]. The molecular mechanisms by which UNC5B/Netrin-1 drive metastasis are unclear. Here, we demonstrate that UNC5B is required for PDAC metastasis and regulates both EMT and Invasion through an SRC-ZEB1 signaling axis. Collectively, these findings establish UNC5B as a central driver of metastatic competence in PDAC, functioning as a molecular integrator of epithelial plasticity and invasive behavior. By linking UNC5B signaling to SRC-ZEB1-dependent regulation of EMT and invasion, UNC5B emerges as a key node that enables tumor cells to acquire and sustain metastatic traits.

## Methods

### Mouse models

Pdx-1-Cre; LSL-Kras^G12D/+^; LSL-Trp53^R172H/+^ (KPC), and (Unc5b^*flox/flox*^) mouse strains on a C57BL/6 background have been described previously [11**–**12]. To generate KPCU mice, Unc5b^*flox/flox*^ animals were crossed with KPC mice to obtain Pdx-1-Cre; LSL-Kras^G12D/+^; LSL-Trp53^R172H/+^; Unc5b^+/+^, ^+/–^, and ^–/–^ generation. Tumor development was monitored by palpation and ultrasound imaging. Mice were euthanized at humane endpoints or when tumors reached institutional size limits, followed by necropsy and collection of primary tumors and organs for molecular and histopathological analyses.

All animal experiments were approved by the University of Rochester Institutional Animal Care and Use Committee and performed in accordance with institutional and national guidelines.

### Cell lines and culture

Human pancreatic ductal adenocarcinoma (PDAC) cell lines (BxPC-3, MIA PaCa-2, SW1990, AsPC-1, PANC-1, KP4) and HEK293T cells were obtained from ATCC or JCRB and authenticated by STR profiling. Met38 murine pancreatic cancer cells were derived from *p48*^cre^; *Kras*^LSL_G12D^; p16^−/−^/*p19*^−/−^ mice. Cells were maintained in DMEM or RPMI supplemented with 10% fetal bovine serum and antibiotics under standard conditions. Mycoplasma testing was performed routinely.

### Orthotopic pancreatic tumor model

Orthotopic pancreatic tumors were established by injection of 5 × 10³ control or *Unc5b*-deficient Met38 cells into the pancreatic tail of anesthetized mice. Tumor progression was monitored, and distal pancreatectomy with splenectomy was performed in selected experiments. Animals were followed for survival and metastatic burden, with humane endpoints applied according to institutional guidelines.

### Histology and immunostaining

Tissues were fixed in formalin, paraffin embedded, and sectioned for hematoxylin and eosin staining or immunohistochemistry. Antigen retrieval, blocking, and antibody incubation were performed using standard protocols. Staining for EMT markers (e.g., ZEB1, SRC, Pancytokeratin, Vimentin, and E-cadherin) was quantified using automated imaging systems. Histopathological assessment, including tumor differentiation, necrosis, and metastasis, was performed in a blinded manner by a board-certified pathologist.

### Immunofluorescence and RNAscope

Immunofluorescence staining was conducted on fixed cells or tissue sections using fluorophore-conjugated antibodies and DAPI nuclear counterstaining. RNA in situ hybridization (RNAscope) was performed on FFPE tissues using target-specific probes, followed by signal amplification and chromogenic detection according to manufacturer protocols.

### Gene manipulation and protein degradation

Gene knockdown and activation were achieved using CRISPR-based systems, including CRISPR interference (CRISPRi) and CRISPR activation (CRISPRa). sgRNAs targeting *UNC5B*, *ZEB1*, and *SRC* were cloned into lentiviral vectors. Lentivirus was produced in HEK293T cells and used to transduce target PDAC cells, followed by antibiotic selection. Gene modulation efficiency was validated by qPCR and Western blotting.

### Protein degradation system

A degron-based system was used to enable inducible degradation of UNC5B. Full-length UNC5B was cloned into a dTAG-compatible expression vector and expressed in PDAC cells. Treatment with the dTAGV-1 compound selectively induced degradation of UNC5B, enabling functional studies of acute protein depletion

### Functional assays

Cell invasion was assessed using Matrigel-coated transwell chambers, and migration was evaluated using wound-healing assays. Cell viability following drug treatment (e.g., dasatinib) was measured using CCK-8 assays. All experiments were conducted with multiple biological replicates.

### RNA sequencing and gene expression analysis

Total RNA was extracted using column-based methods and subjected to high-throughput sequencing. Reads were quality-checked and quantified using Salmon, and differential expression analysis was performed using EdgeR. Pathway enrichment analysis was conducted using GAGE and ShinyGO, with false discovery rate (FDR) correction applied. EMT scores were calculated from normalized expression data.

### Quantitative PCR and Western blotting

Gene expression was quantified using TaqMan-based qPCR with normalization to housekeeping genes. Protein expression was analyzed by western blot using validated antibodies against EMT markers and signaling proteins, with chemiluminescent detection.

### Statistical analysis

Data are presented as mean ± s.d. Statistical significance was determined using unpaired two-tailed Student’s *t* tests or multiple comparison tests with FDR correction where appropriate. Survival analyses were performed using Kaplan-Meier methods with log-rank testing. Analyses were conducted using GraphPad Prism, with *P* < 0.05 considered significant. Detailed methodologies are provided in Supplementary Methods File 1.

## Results

### UNC5B is highly expressed in PDAC and is a driver of metastatic progression

**Fig. 1:**
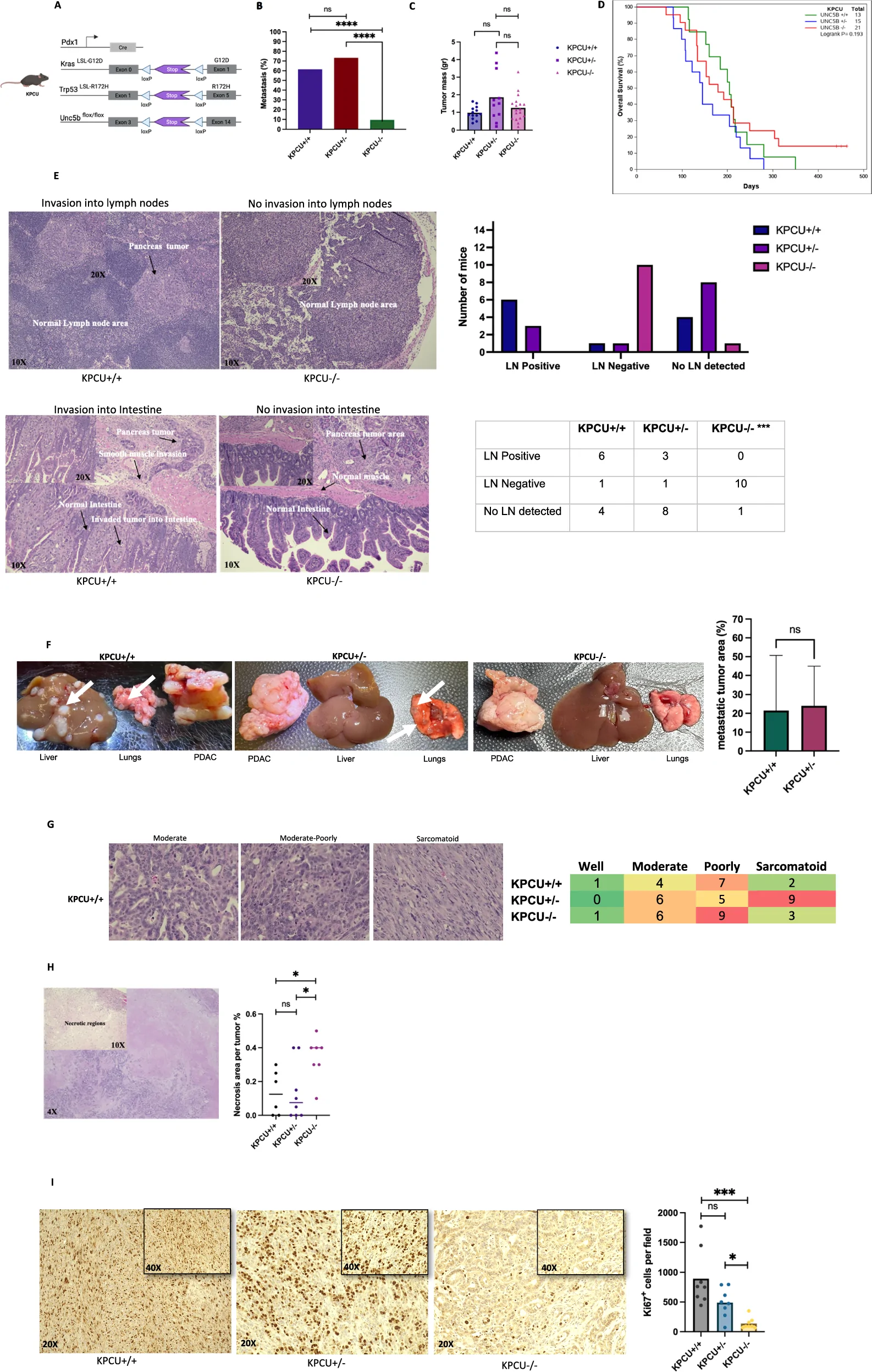
Tumor progression, survival, histopathology, and metastasis in the KPCU mouse model. **A** Schematic representation of genetically engineered mouse models (KPCU) for Pancreatic Cancer. **B** Metastatic tumors developed in 73.3% of KPCU^+/–^ (11/15) and 61.5% of KPCU^+/+^ (8/13) mice, whereas only 8.7% of KPCU^–/–^ mice (2/23) exhibited metastases. Statistical analysis using Fisher’s exact test revealed a highly significant difference between KPCU^-/-^ and the other genotypes (****P = 0.0001). **C** Tumor growth rates were measured in KPCU^+/+^, KPCU^+/-^, and KPCU^-/-^ mice. No significant differences were detected between genotypes by Welch’s *t* test (Satterthwaite correction): KPCU–/– vs KPCU^+/−^, *P* = 0.2140; KPCU^–/–^ vs KPCU^+/+^, *P* = 0.1820; KPCU^+/−^ vs KPCU^+/+^, *P* = 0.0701. **D** Kaplan-Meier survival analysis revealed no statistically significant differences in overall survival among the three genotypes (*P* = 0.193, log-rank [Mantel-Cox] test). **E** Hematoxylin and eosin (H&E) images of primary pancreatic tumors from genetically engineered mice with Unc5b expression (KPCU^+/+^, ^+/–^) or without (KPCU^–/–^). Unc5b-positive tumors exhibited invasion into surrounding smooth muscle (arrows), intestinal wall (arrow), and lymph nodes (LNs)(arrows). In contrast, KPCU^–/–^ (Unc5b-deleted) tumors showed no evidence of smooth muscle or intestinal invasion and lymph node invasion, as the tumor remains confined to the pancreas. Pearson’s Chi-square test revealed a significant difference in the incidence of positive lymph nodes among KPCU groups (χ² = 14.44, df = 2, ***P = 0.0004), with estimated positivity rates of 85.7%, 75.0%, and 0% for the (+/+), ( + /–), and (–/–) groups, respectively. Grouping (+/+) and (+/-) mice, based on similar positivity rates, revealed a significant difference versus (–/–) mice by Fisher’s exact test (***P = 0.0002). **F** Representative gross images of endpoint necropsies show that KPCU^+/+^ and KPCU^+/–^ mice developed visible metastatic lesions in both liver and lungs, whereas KPCU^-/-^ mice exhibited no detectable metastases. **G** H&E staining of primary tumors revealed no significant histopathological differences among KPCU^+/+^, KPCU^+/–^, and KPCU^–/–^ mice. Tumors retained moderate, poorly, and sarcomatoid features irrespective of Unc5b status. Statistical analysis by Pearson’s chi-square test confirmed no significant association between genotype and histopathological features (χ² = 7.27, df = 6, exact *P* = 0.3101). **H** Representative H&E-stained sections showing areas of tumor necrosis across different KPCU genotypes. KPCU^–/–^ (Unc5b deficient) tumors exhibited significantly greater necrotic regions compared to KPCU^+/+^ and KPCU^+/–^ tumors. Quantification of necrotic area is shown on the right. **I** Immunohistochemistry (IHC) staining of Ki67 in primary pancreatic tumor tissues from KPCU mice demonstrated that tumors with intact Unc5b exhibited higher proliferative activity, whereas tumors from mice lacking Unc5b alleles showed a marked reduction in Ki67-positive cells. Ki67 expression was quantified by IHC in primary tumor tissues from three groups of KPCU mice (KPCU^+/+^, KPCU^+/−^, KPCU^−/−^), with 8 mice per group analyzed. Pairwise comparisons between groups were conducted using the Wilcoxon rank-sum test. A statistically significant reduction in Ki67 expression was observed between KPCU^+/+^ and KPCU^–/–^ tumors (***P = 0.0009) and between KPCU^+/–^ and KPCU^–/–^ tumors (*P = 0.0116), indicating reduced tumor cell proliferation upon loss of Unc5b. No statistically significant difference was observed between KPCU^+/+^ and KPCU^+/–^ tumors (*P* = 0.0831).

The Pdx-1-Cre; LSL-Kras^G12D/+^;LSL-Trp53^R172H/+^ (KPC) genetically engineered mouse model recapitulates key features of PDAC tumor progression, including the development of regional lymph node and distant metastases [11]. To investigate the functional role of the Unc5b receptor in PDAC development and metastasis, we generated mice harboring a conditional knockout allele of Unc5b (*Unc5b*^*flox/flox*^) [13]. This approach allowed us to assess the consequences of Unc5b loss in the context of KRAS-driven pancreatic tumorigenesis and its contribution to metastatic potential [14, 15]. The resulting KPCU mice were born at expected Mendelian ratios and exhibited no overt developmental or functional abnormalities of the pancreas (Fig. 1A).

We examined tumor growth, metastatic burden, survival, and histopathology across KPCU mice harboring zero, one, or two functional alleles of Unc5b when mice developed endpoint criteria for euthanasia. Loss of Unc5b expression in the primary tumor was confirmed by in situ hybridization technique (Fig. S1A). Metastatic disease was observed in 73.3% of KPCU^+/–^ and 61.5% of KPCU^+/+^ mice, whereas 0% of the KPCU^–/–^ mice developed detectable metastases (Fig. 1B). Notably, two mice classified as KPCU^–/–^ (8.7%) developed metastatic lesions but retained Unc5b expression in their primary tumors, consistent with incomplete Cre-LoxP recombination and indicating that the system is not fully efficient (Fig. S1B) [16]. Primary tumor weights were similar among KPCU^+/+^, KPCU^+/–^, and KPCU^–/–^ mice indicating the loss of Unc5b did not impair primary tumorigenesis (Fig. 1C). Consistent with this finding, overall survival did not differ significantly across genotypes, potentially reflecting the fact that all mice ultimately succumbed to primary tumor burden, frequently accompanied by gastrointestinal and biliary obstruction (Fig. 1D) [17]. Histopathological examination by a Gastrointestinal (GI) pathologist of primary pancreatic tumors from KPCU^+/+^ and KPCU^–/–^ mice revealed striking differences in invasive behavior. H&E-stained sections from KPCU^+/+^ tumors showed clear evidence of local invasion into small intestinal smooth muscle and epithelial layers. In addition, tumor cells were detected within regional lymph nodes, consistent with early lymphatic dissemination. In contrast, tumors from KPCU^-/-^ mice remained well confined within the pancreatic parenchyma, with no observable invasion into the small intestine or lymphatic structures. These findings indicate that Unc5b expression is required for local tissue invasion and lymph node metastasis in pancreatic ductal adenocarcinoma (Fig. 1E). At necropsy, macroscopic metastases in liver and lung were evident in KPCU^+/+^ and KPCU^+/–^ mice but absent in KPCU^–/–^ animals (Fig. 1F). Histological analysis of primary tumors revealed no significant differences in tumor differentiation or architecture among genotypes, with all exhibiting a spectrum of histologies from well-differentiated to moderately or poorly differentiated to sarcomatoid as has been described for this model (Fig. 1G) [18]. Interestingly, primary pancreatic tumors across KPCU genotypes revealed marked differences in both necrosis and proliferative capacity. Tumors from KPCU^–/–^ mice exhibited significantly larger areas of necrosis compared to those from KPCU^+/+^ and KPCU^+/–^ mice, suggesting compromised tumor cell viability upon loss of Unc5b. Consistent with this observation, Ki67 immunostaining revealed a reduction in proliferative activity in KPCU^–/–^ tumors relative to controls (Fig. 1H, I). This reduction in proliferation was further supported in vitro, where *UNC5B* knockdown in the Met38 pancreatic cancer cell line led to decreased Ki67 expression, as assessed by immunocytochemistry (ICC) (Fig. S1C).

To further assess the role of Unc5b in vivo we used a murine pancreatic cell line (Met38) derived from a hepatic metastasis generated by orthotopic implantation of a cell line derived from p48^cre^; Kras^LSL_G12D^; p16^−/−^/p19^−/−^ (Ink4a.1) [9]. Mice were implanted with Met 38 Unc5b wild type (WT) or Unc5b knock down (KD) cells orthotopically and approximately two weeks later primary tumors were resected. The Unc5b KD group exhibited a complete absence of metastatic lesions, in contrast to widespread metastases observed in the control group, confirming a critical role for Unc5b in metastatic progression (Fig. S1D). We next examined the expression of UNC5B in human PDAC using publicly available data sets. Using TCGA data we found that a majority of PDAC patients expressed UNC5B with an average of protein-coding Transcripts Per Million (pTPM) of 20.2. In a separate cohort of both human primary and metastatic PDAC we observed that UNC5B mRNA was highly expressed. We further analyzed a public dataset of 45 matched pairs of human PDAC tumors and adjacent non-tumor pancreatic tissues in which normal pancreas tissues express a low level of UNC5B compared to adjacent tumor tissues (Fig. S1E,F, G). Likewise, we observed the same observation in our KPCU mouse models in which mouse PDAC tumors express a high level of Unc5b while normal pancreas showed no-low expression of Unc5b (Fig. S1H). Collectively, we identify UNC5B as highly expressed in PDAC and as a driver of metastatic tumor progression.

### UNC5B regulates the EMT program in PDAC to promote a mesenchymal phenotype

**Fig. 2:**
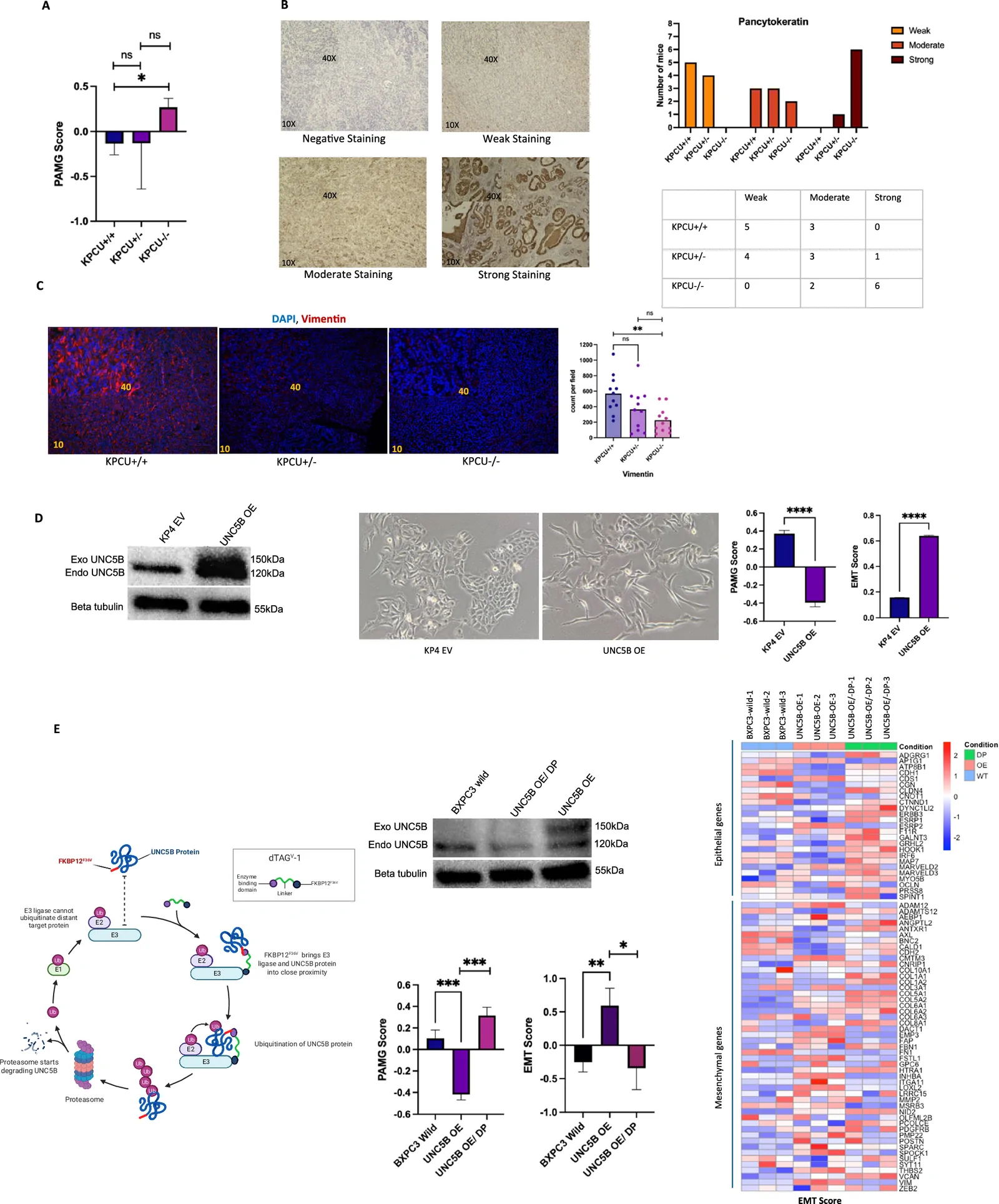
UNC5B Induces a Mesenchymal Phenotype in PDAC and Modulates the EMT Program. **A** PAMG (Pancreatic Adenocarcinoma Molecular Gradient) scores were calculated from RNA-seq data of primary tumors isolated from KPCU^+/+^ (wild-type), KPCU^+/–^, and KPCU^–/–^ mice. KPCU^–/–^ tumors displayed significantly higher PAMG scores compared to wild-type tumors, suggesting a shift toward a more epithelial-like, less aggressive phenotype. **B** Loss of Unc5b is significantly associated with increased epithelial marker expression, as assessed by Pan-cytokeratin (AE1/AE3) immunohistochemistry. Quantitative scoring of staining intensity across three genotypes (KPCU^+/+^, KPCU^+/–^, and KPCU^–/–^) revealed a progressive shift from predominantly weak or moderate staining in wild-type and heterozygous tissues to strong staining in Unc5b deficient samples. Chi-square analysis of categorical staining data revealed a significant association between KPCU genotype and staining intensity (χ² = 13.77, df = 4, *P* = 0.0081 [asymptotic]; exact **P = 0.0054), supporting the non-random distribution of stronger staining in Unc5b deficient tissues. **C** Immunofluorescence (IF) staining for Vimentin, a canonical mesenchymal marker, was performed on primary tumor tissues from KPCU mice. Quantification revealed significantly higher Vimentin expression in KPCU^+/+^ (Unc5b wild-type) tumors compared to KPCU^–/–^ tumors, indicating enhanced mesenchymal features in the presence of Unc5b. Statistical analysis showed a significant difference between groups (*P < 0.05). **D** Western blot analysis (left) confirms exogenous UNC5B overexpression in KP4 pancreatic cancer cells. Overexpression (OE) of exogenous UNC5B in human epithelial-like KP4 pancreatic cancer cells induced a phenotypic shift toward a mesenchymal state. This transition was supported by transcriptomic analysis using RNA-seq, which revealed a significantly reduced PAMG score in UNC5B-overexpressing cells, consistent with mesenchymal phenotype change in UNC5B OE group. **E** Schematic illustration of Degron-mediated UNC5B protein degradation. A mutant FKBP^12^F36V^ degron tag was fused to the C-terminus of the exogenous UNC5B gene, enabling conditional protein degradation. Cells stably expressing UNC5B-FKBP^12^F36V^ produce a functional, tagged UNC5B protein that remains stable under normal conditions. Upon addition of the dTAG^V^-1 small molecule degrader, the dTAG^V^-1 domain recruits FKBP^12^F36V^ tagged UNC5B and an E3 ligase complex, leading to ubiquitination and subsequent proteasomal degradation of the UNC5B fusion protein. Created with BioRender.com. Western blot analysis confirmed robust degradation of UNC5B following dTAG^V^-1 treatment (UNC5B OE/ DP). EMT status was assessed in BxPC-3 wild, OE, and DP models using an EMT gene signature comprising established epithelial and mesenchymal markers. The MAK EMT Score was calculated as the average expression difference between mesenchymal and epithelial genes, with higher scores indicating a more mesenchymal phenotype. UNC5B OE induced a mesenchymal-like phenotype, while acute UNC5B depletion (UNC5B OE/ DP) in the same cells reverted them to the baseline or more epithelial-like state. This phenotypic shift was validated by transcriptomic analysis using RNA-seq, where both PAMG and EMT scoring systems demonstrated a transition from a mesenchymal to epithelial phenotype upon UNC5B degradation.

To evaluate the impact of UNC5B on EMT, we analyzed EMT-associated gene signatures in primary tumors from KPCU mice using both the MAK and Pancreatic Adenocarcinoma Molecular Gradient (PAMG) scoring systems [19, 20]. The MAK EMT score quantifies EMT status based on the expression of 77 defined genes comprised of 55 mesenchymal and 22 epithelial genes, where higher scores correspond to a more mesenchymal phenotype and lower scores indicate a more epithelial-like state. While this score has been validated across eleven different human solid organ cancers, PDAC is not one of them. In contrast, the PAMG system is a comprehensive molecular scoring method that considers the expression of all genes to define the EMT spectrum in PDAC, with higher scores reflecting a more epithelial, less aggressive phenotype and lower scores indicating a mesenchymal, more aggressive program. To assess transcriptional programs associated with epithelial-mesenchymal dynamics, we applied both the PAMG and MAK scoring systems to KPCU tumor transcriptomes. KPCU^–/–^ tumors exhibited significantly elevated PAMG scores compared to wild-type controls, indicating a shift toward a more epithelial transcriptional state (Fig. 2A). While the MAK EMT score trended lower in KPCU^–/–^ tumors, this difference did not reach statistical significance (Fig. S2A). In parallel, Immunohistochemistry for Pan-cytokeratin (AE1/AE3) revealed a genotype-dependent increase in epithelial staining, with KPCU^–/–^ tissues showing predominantly strong staining compared to weak or moderate staining in KPCU^+/+^ and KPCU^+/–^ tumors. Quantitative scoring of staining intensity across genotypes demonstrated a progressive shift toward higher epithelial marker expression with Unc5b loss (Fig. 2B). This observation was corroborated by double immunostaining for E-cadherin and Vimentin, which demonstrated elevated Vimentin expression in KPCU^+/+^ and KPCU^+/-^ tumors, whereas KPCU^–/–^ tumors retained strong E-cadherin expression and exhibited minimal Vimentin staining (Fig. S2B). Consistently, immunofluorescence for the mesenchymal marker Vimentin revealed significantly higher expression in KPCU^+/+^ tumors relative to KPCU^–/–^ tissues, indicating that Unc5b presence reinforces mesenchymal features in PDAC (Fig. 2C). Together, these data demonstrate that Unc5b plays a pivotal role in maintaining mesenchymal features in murine PDAC, whereas its loss shifts tumor cells toward an epithelial-like state, consistent with the transcriptional reprogramming observed in PAMG scoring.

To determine whether UNC5B directly regulates epithelial-mesenchymal plasticity in human PDAC cells, we modulated UNC5B expression in cell lines with distinct epithelial-like or mesenchymal phenotypes. Overexpression of UNC5B in epithelial-like KP4 cells induced a striking phenotypic shift toward a mesenchymal state, characterized by elongated, spindle-shaped morphology, loss of cell-cell adhesion, and increased cellular scattering. Transcriptomic analysis by RNA-seq supported this transition, revealing a significantly reduced PAMG score and increase in MAK EMT score in UNC5B-overexpressing cells, consistent with acquisition of mesenchymal features (Fig. 2D). Consistently, immunocytochemistry and Western blot analyses revealed robust upregulation of Vimentin in UNC5B-overexpressing KP4 cells, further supporting the transition toward a mesenchymal, invasive phenotype. In contrast, UNC5B depletion increased E-cadherin expression, suggesting reversion toward an epithelial state and suppression of EMT. (Fig. S2C). Conversely, reducing the expression of UNC5B using a CRISPR/Cas9 system in mesenchymal-like MIA PaCa-2 cells promoted epithelial-like characteristics, including a more compact, cobblestone morphology and restoration of cell-cell contacts. In parallel, we employed a CRISPR activation (CRISPRa) system to selectively upregulate endogenous UNC5B expression in MIA PaCa-2 cells. This approach allowed us to enhance UNC5B levels while minimizing potential off-target effects associated with conventional exogenous overexpression strategies, thereby providing a more physiologically relevant model to assess the functional impact of UNC5B upregulation. Transcriptomic analysis using both PAMG and MAK EMT scoring systems revealed a pronounced mesenchymal phenotype in these cells, further confirming that elevated endogenous UNC5B levels are sufficient to reinforce mesenchymal characteristics (Fig. S2D). To extend our findings beyond human PDAC cells, we employed our murine Met38 Unc5b KD cell line. Consistent with observations in human cell lines, Unc5b-KD Met38 cells exhibited a shift toward an epithelial-like state and reduced Vimentin expression. Transcriptomic analysis confirmed this phenotypic transition, with significantly higher PAMG scores compared to control cells (Fig. S2E). Together, these complementary gain- and loss-of-function studies establish UNC5B as a critical regulator of epithelial-mesenchymal plasticity in both human and murine PDAC, driving both morphological and transcriptional transitions between epithelial and mesenchymal states.

To further validate these findings, we developed a targeted protein-degradation strategy using a Degron system. In this approach, the UNC5B gene was fused to the N-terminus of a mutated FKBP^12^F36V^ tag and transduced into human BxPC-3 cells. Upon treatment with a dTAG^V^-1 compound, one end of the molecule specifically binds the mutated FKBP^12^F36V^ tag present only on the exogenous UNC5B, while the other recruits the E3 ubiquitin ligase complex and associated ubiquitination machinery, leading to proteasomal degradation of the tagged UNC5B protein (Fig. 2E). This system allows selective and inducible depletion of UNC5B, enabling functional interrogation of its role in EMT.

We successfully generated stable human PDAC cell lines incorporating the Degron system to enable inducible and selective UNC5B degradation. Western blot analysis confirmed robust and specific depletion of exogenous UNC5B in human BxPC-3 upon dTAG^V^-1 treatment (Fig. 2E). Functionally, UNC5B overexpression induced a pronounced mesenchymal-like phenotype in BxPC-3 cells, while acute UNC5B depletion in the same cells triggered a rapid phenotypic reversion toward a more epithelial-like state. This phenotypic switch was corroborated at the transcriptomic level by RNA-seq, where both PAMG and MAK EMT scoring systems consistently demonstrated a transition from a mesenchymal to epithelial program upon UNC5B degradation (UNC5B OE/DP) (Fig. 2E). To assess EMT dynamics across conditions, we performed unsupervised hierarchical clustering of canonical EMT-associated genes and visualized their expression patterns as a heatmap. The analysis revealed pronounced transcriptional heterogeneity driven by UNC5B modulation, indicating condition-specific regulation of EMT programs. Mesenchymal-associated genes, including *VIM*, *ADAM12*, *CMTM3*, *EMP3*, *FSTL1*, and *LOXL2*, were broadly upregulated in UNC5B overexpression (UNC5B OE) samples, whereas epithelial markers such as *CDH1*, *CLDN4*, *CTNND1*, *IRF6*, *GRHL2*, and *MAP7* were concomitantly downregulated. Notably, acute depletion of UNC5B (UNC5B OE/ DP) in the same cellular context reversed these transcriptional changes, restoring mesenchymal- and epithelial-associated gene expression toward baseline levels. Together, these data indicate that UNC5B acts as a key regulator of EMT-associated transcriptional plasticity (Fig. 2E).

Pathway enrichment analysis of hallmark upregulated genes revealed robust activation of signaling programs associated with cell motility, invasion, and mesenchymal transition in UNC5B-expressing cells. Among the most significantly enriched pathways were EMT, ECM-receptor interaction, IL-2/STAT signaling, endothelial cell development, basal cell carcinoma, hypoxia, and axon guidance. Notably, activation of these pathways was reversed upon acute depletion of UNC5B in the same cellular context, indicating that their regulation is UNC5B dependent. In contrast, analysis of hallmark downregulated genes revealed suppression of immune-related pathways, including type II interferon receptor signaling, negative regulation of viral processes, and interferon-γ and interferon-α responses in UNC5B-overexpressing cells. Importantly, these transcriptional changes were also reversed following UNC5B depletion, indicating that UNC5B expression is associated with coordinated activation of pro-invasive programs and attenuation of interferon-mediated signaling. These transcriptomic alterations are consistent with the observed phenotypic shift toward a mesenchymal and motile state, underscoring UNC5B as a key regulator of tumor cell plasticity and invasive potential (Fig. S2F).

### UNC5B promotes epithelial-mesenchymal transition through SRC and ZEB1 regulation

**Fig. 3:**
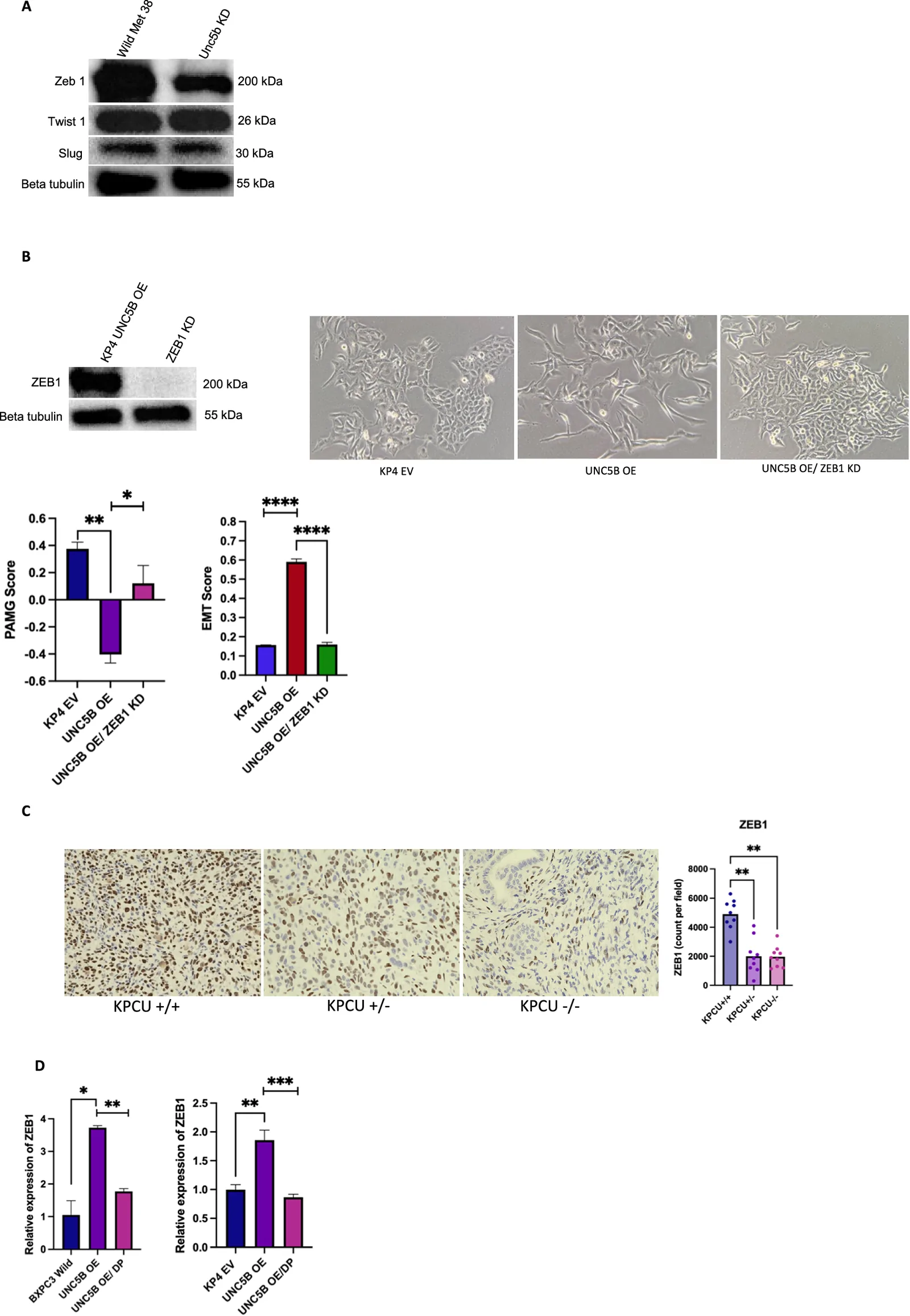
UNC5B facilitates epithelial-mesenchymal transition through ZEB1 regulation. **A** Western blot analysis of EMT-inducing transcription factors in murine Met38 cells following Unc5b knockdown. Among the assessed factors, only Zeb1 expression was notably reduced upon Unc5b KD, while Twist1 and Slug levels remained unchanged. Snail protein was undetectable in this cell line under the tested conditions. **B** Western blot analysis shows ZEB1 KD in human KP4 cells overexpressing UNC5B (UNC5B OE). ZEB1 KD reversed the mesenchymal phenotype of KP4 cells (UNC5B OE) toward an epithelial-like phenotype, as determined by MAK EMT and PAMG scoring system from RNA-seq analysis. Statistical comparisons between the parental cell line and each OE and KD were performed using unpaired, two-tailed *t* tests. **C** Immunohistochemistry (IHC) staining of Zeb1 in primary pancreatic tumor tissues from KPCU mice revealed that tumors with intact Unc5b exhibited higher Zeb1 levels, while tumors from mice lacking one or both Unc5b alleles showed a marked reduction in Zeb1 expression. Zeb1 expression was quantified by IHC in primary tumor tissues from three groups of KPCU mice (KPCU^+/+^, KPCU^+/–^, KPCU^–/–^), with a total of 9 tissue slides analyzed per group. Pairwise comparisons between groups were conducted using the Wilcoxon Rank Sum test. Statistically significant differences in ZEB1 expression were found between KPCU^+/+^ and KPCU^+/−^ (**P = 0.0011) and between KPCU^+/+^ and KPCU^-/-^ (**P = 0.0006). **D** qPCR analysis shows Human BxPC-3 and KP4 cells overexpressing UNC5B exhibited elevated ZEB1 expression at the RNA level. Acute depletion of UNC5B using the dTAG^V^-1 degrader compound led to a marked reduction in ZEB1 expression in the same cells.

Recently deletion of the canonical EMT transcription factor gene, ZEB1 significantly reduced precursor lesion formation, local invasion, and distant metastasis in a murine model; however, the mechanism that regulates Zeb1 remains unknown [8]. We examined the effect of UNC5B KD or OE on the levels of several canonical EMT transcription factors, including Zeb1, Snail1, Slug, and Twist1. Knockdown of Unc5b in the murine PDAC cell line Met38 resulted in a marked reduction of Zeb1 protein levels, while the expression Snail1, Slug, and Twist1, remained largely unchanged (Fig. 3A). Similarly, UNC5B knockdown in the human PDAC cell line MIA PaCa-2 led to a robust decrease in ZEB1 expression without affecting the levels of other canonical EMT transcription factors. However, TWIST and SLUG were undetectable in MIA PaCa-2 cells under both control and UNC5B KD conditions (Fig. S3A). In parallel, overexpression of UNC5B in the KP4 human PDAC cell line led to a significant upregulation of ZEB1, while TWIST and SLUG remained undetectable (Fig. S3B). These findings indicate that UNC5B is an upstream regulator of ZEB1. To determine whether the UNC5B mediated effects on EMT are functionally due to ZEB1, we knocked down ZEB1 in UNC5B-overexpressing KP4 cells using a CRISPR interference (CRISPRi) system. First, we examined cell morphology and found that UNC5B overexpression led to a more mesenchymal phenotype that was reversed upon ZEB1 KD. Moreover, we examined the MAK EMT and PAMG scores of these cells and found that the decrease in PAMG score and increased MAK EMT score induced by UNC5B OE was also reversed upon ZEB1 KD (Fig. 3B). To validate these observations in vivo, we performed immunohistochemistry staining for Zeb1 in primary pancreatic tumor tissues from KPCU mice. Tumors from mice with intact Unc5b (KPCU^+/+^) exhibited higher Zeb1 levels, whereas tumors from mice lacking one or both Unc5b alleles (KPCU^+/–^ and KPCU^–/–^) showed a marked reduction in Zeb1 expression (Fig. 3C). Altogether, these data establish UNC5B as a critical upstream regulator of ZEB1 in PDAC, both in vitro and in vivo. These results indicate that ZEB1 is essential for UNC5B-mediated induction of EMT, demonstrating that the pro-mesenchymal effects of UNC5B in PDAC cells are largely dependent on ZEB1. This establishes ZEB1 as a critical downstream effector through which UNC5B drives cellular plasticity and the mesenchymal phenotype.

To further determine the specific role of the UNC5B receptor and to rule out potential off-target effects associated with gene editing approaches, we employed our degron-based protein degradation system. Human BxPC-3 cells overexpressing UNC5B exhibited a fourfold increase in ZEB1 expression. However, upon treatment with the dTAG^V^-1 compound, which induces targeted degradation of the UNC5B protein via the degron system, ZEB1 levels decreased by approximately twofold, returning to baseline, while levels of other EMT-inducing transcription factors remained largely unchanged (Fig. 3D & S3C). The same changes in ZEB1 level were observed in human KP4 cells in which UNC5B OE led to upregulation of ZEB1 and UNC5B depletion using dTAG^V^-1 compound reverted the ZEB1 level to baseline (Fig. 3D & S3D). These findings suggest that UNC5B is both necessary and sufficient to drive ZEB1 expression in PDAC cells and its regulatory effect on ZEB1 is reversible and directly dependent on UNC5B protein levels.

**Fig. 4:**
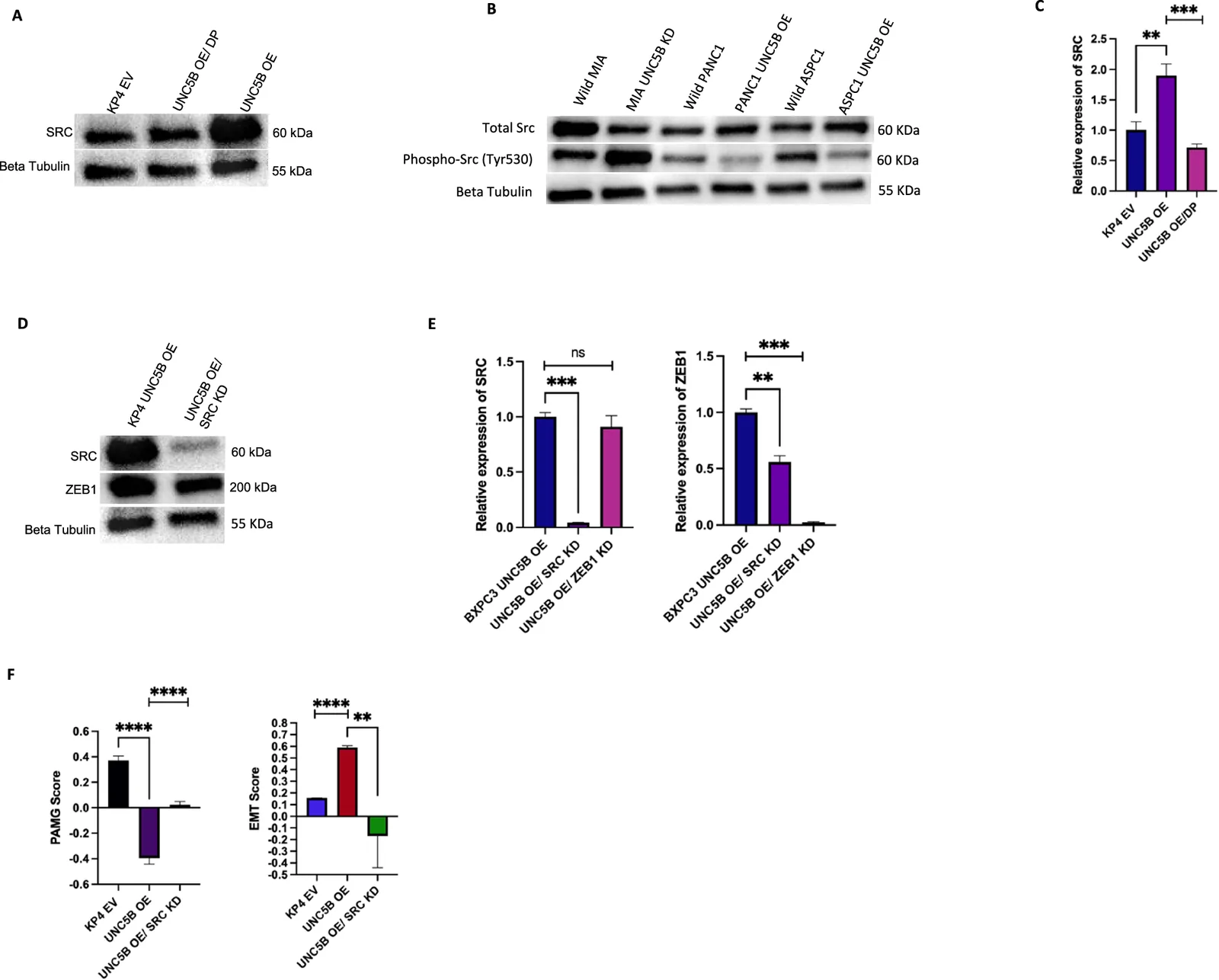
UNC5B Promotes ZEB1-driven epithelial-mesenchymal transition through SRC signaling. **A** Western blot analysis shows that UNC5B OE in KP4 cells leads to increased levels of total SRC. Upon treatment with the dTAG^V^-1 compound to acutely deplete UNC5B (UNC5B OE/ DP), SRC level returned to baseline. **B** Western blot analysis of UNC5B KD and overexpression in different human PDAC cell lines revealed that UNC5B modulates SRC activity. UNC5B KD led to reduced total SRC protein levels and a concurrent increase in phosphorylation at the inhibitory Tyr530 site, indicative of decreased SRC activity. Conversely, UNC5B overexpression increased total SRC levels and reduced Tyr530 phosphorylation. **C** qPCR analysis shows that UNC5B OE in KP4 cells leads to increased levels of total SRC. Upon UNC5B depletion (UNC5B OE/ DP) using dTAG^V^-1 compound, SRC level returned to baseline. **D** Western blot analysis shows knockdown of SRC in KP4 UNC5B OE cells resulted in a reduction of ZEB1 expression. **E** qPCR analysis shows knockdown of SRC in BxPC-3 UNC5B OE cells significantly reduced ZEB1 levels, whereas knockdown of ZEB1 did not alter SRC expression, suggesting that SRC acts upstream of ZEB1 in the UNC5B signaling axis. **F** EMT analysis shows SRC KD reversed the mesenchymal status of KP4 cells (UNC5B OE/SRC KD) toward an epithelial-like, as determined by MAK EMT and PAMG scoring system from RNA-seq analysis. Statistical comparisons between the parental cell line and each OE and KD were performed using unpaired, two-tailed *t* tests.

To investigate the signaling mechanisms downstream of UNC5B and how it regulates ZEB1, we examined SRC kinase expression and activation status in multiple human PDAC cell lines. We selected SRC as a candidate mediator because prior work demonstrated that SRC-1 physically interacts with UNC5 family and mediates its receptor tyrosine phosphorylation [21]. It has been shown that phosphorylation at Tyr530 decreases SRC activity [22]. We found UNC5B overexpression not only increases total SRC levels but also decreases the phosphorylation at Tyr530 (Fig. 4A, B, & S3E). We next examined SRC mRNA levels by qPCR using KP4 cells with UNC5B empty vector and overexpression and found that overexpression led to increased levels that decreased with UNC5B depletion indicating that UNC5B transcriptionally regulates SRC (Fig. 4C). Taken together UNC5B regulates SRC both transcriptionally and post-translationally.

To investigate how UNC5B regulates SRC post-translationally, we examined the PTEN phosphatase which is a known negative regulator of SRC [23]. Using BxPC3 cells with UNC5B overexpression or depletion, we observed that total PTEN levels decreased upon UNC5B overexpression and increased following UNC5B depletion, suggesting that PTEN may contribute to UNC5B-mediated post-translational regulation of SRC signaling (Fig. S3F).

To investigate whether SRC functions upstream of ZEB1, we performed knockdown experiments in UNC5B-overexpressing KP4 cells. Silencing SRC led to a marked reduction in ZEB1 levels, indicating a regulatory link (Fig. 4D). This observation was further validated in a second human PDAC cell line, BxPC-3, where SRC knockdown similarly reduced ZEB1 expression, whereas ZEB1 knockdown had no effect on SRC levels (Fig. 4E). These findings suggest that SRC acts upstream of ZEB1 and may directly or indirectly regulate its expression in pancreatic cancer cells. To further assess the functional relevance of this axis, we evaluated EMT and differentiation states using two transcriptomic scoring systems: the MAK EMT score and the PAMG score. Both SRC and ZEB1 knockdown in UNC5B-overexpressing cells resulted in a decrease in the MAK EMT score and a concomitant increase in the PAMG score, indicative of a shift toward a more differentiated, epithelial-like phenotype (Fig. 4F).

### UNC5B Promotes PDAC Invasion through the SRC-ZEB1 Signaling Axis

**Fig. 5:**
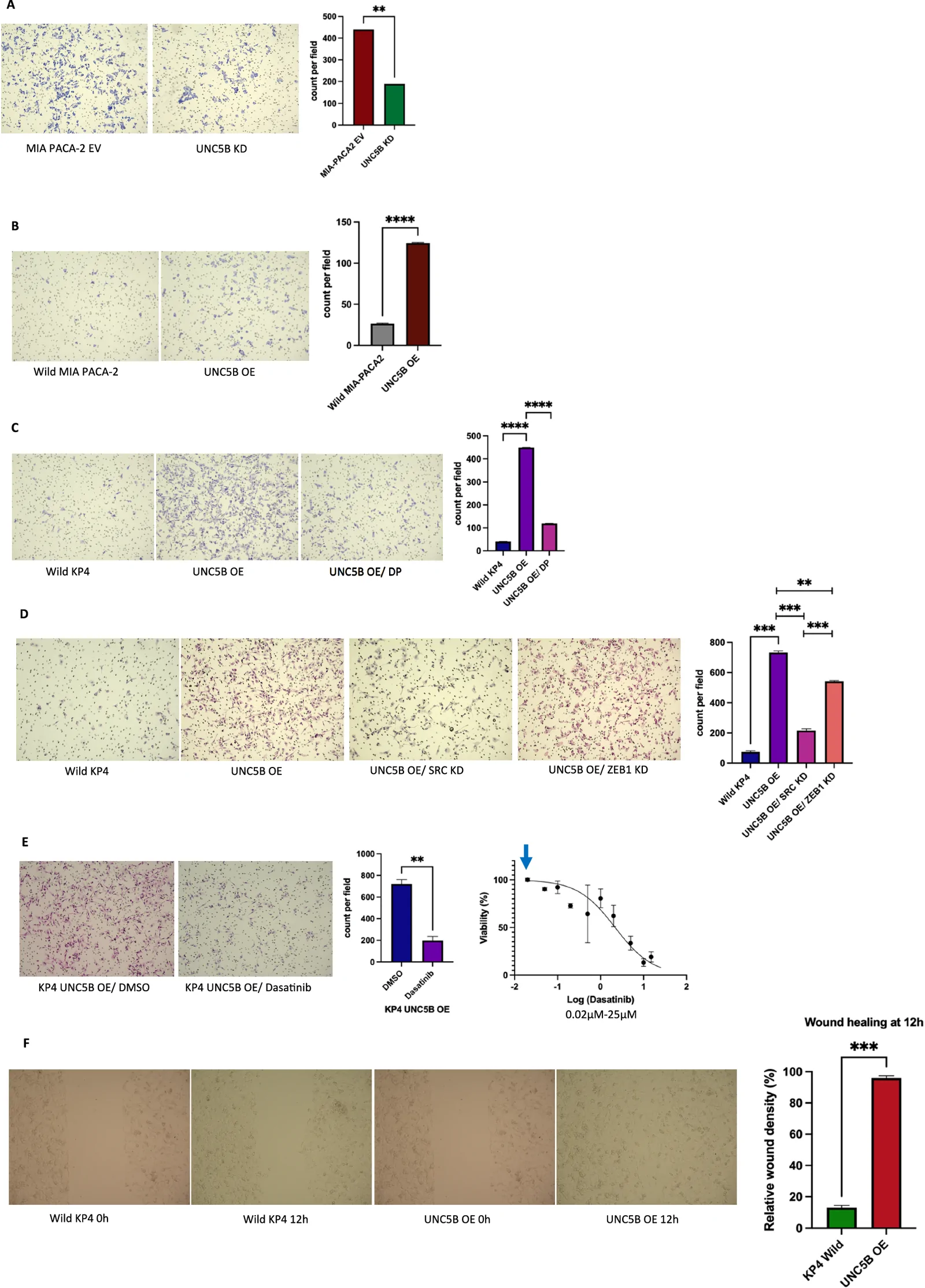
UNC5B promotes the invasion of PDAC through SRC-ZEB1 axis. **A** Transwell invasion assays were performed using Matrigel-coated inserts to assess invasive potential of MIA PaCa-2 cells. UNC5B KD significantly reduced invasive potential compared to control cells. A total of 40,000 cells were seeded per insert, cells were fixed and stained after 22-24 h incubation. **B** In contrast, Cells overexpressing endogenous UNC5B (UNC5B OE) displayed significantly increased invasion compared to control cells. A total of 20,000 cells were seeded per insert, and invaded cells were fixed, stained, and quantified after 22–24 h incubation. **C** Representative image of Transwell invasion assay showing the effects of exogenous UNC5B OE and depletion (UNC5B OE/ DP) using a protein degrader system in KP4 cells. In the UNC5B OE model, cells exhibited enhanced invasive capacity. Treatment with the dTAG^V^-1 compound induced targeted degradation of UNC5B in UNC5B OE group, resulting in reduced invasion compared to the vehicle-treated (negative dTAG) control group. **D** Transwell invasion assays in KP4 UNC5B OE cells show that knockdown of SRC or ZEB1 significantly reduces invasive capacity. SRC knockdown led to a greater reduction in invasion compared to ZEB1 knockdown. **E** Dasatinib reduced the invasion capacity of KP4 UNC5B OE cells by inhibiting SRC activity. Cells were treated with Dasatinib at 0.02 μM for 22–24h. **F** Wound healing assay showing that UNC5B OE cells exhibit accelerated gap closure compared to wild-type KP4 cells at 12 h. 20,000 cells were seeded per well, and images were acquired at the indicated time points following wound generation. Data represent mean ± SEM from three independent biological replicates; *P* values were calculated using an unpaired two-tailed *t* test.

To investigate the functional role of UNC5B in pancreatic cancer cell invasion, we performed Transwell Matrigel invasion assays using MIA PaCa-2 and KP4 cells. In MIA PaCa-2 cells, UNC5B KD significantly impaired invasive capacity, while overexpression of endogenous UNC5B using CRISPR Activation system significantly increased invasive capacity compared to control cells (Fig. 5A, B). To further validate the role of UNC5B in invasion, we used our chemical degrader approach, Degron system, in KP4 cells stably expressing UNC5B. Acute depletion of UNC5B protein resulted in a marked reduction in invasion relative to vehicle-treated (dTAG-negative compound) controls, confirming that UNC5B promotes invasion in a reversible and targetable manner (Fig. 5C). We next interrogated the molecular mediators of UNC5B-induced invasion. In KP4 cells overexpressing UNC5B, knockdown of either SRC or ZEB1 significantly impaired invasive capacity, with SRC knockdown producing a more pronounced effect than ZEB1 knockdown (Fig. 5D). Pharmacological inhibition of SRC with Dasatinib recapitulated the phenotype observed in SRC knockdown cells, leading to a comparable reduction in invasive capacity (Fig. 5E). In parallel, we performed a wound healing assay to assess the migratory and invasive behavior of PDAC cells following UNC5B modulation. UNC5B overexpression significantly enhanced cell migration, with complete wound closure observed after 12 h of incubation compared to the control group (Fig. 5F). Collectively, these results indicate that UNC5B can engage both SRC and ZEB1 signaling to promote invasion.

## Discussion

Our findings establish UNC5B as a central mediator of metastatic dissemination in pancreatic ductal adenocarcinoma. Using a genetically engineered KPC mouse model with pancreas-specific Unc5b deletion, we found that loss of Unc5b completely abrogates metastatic spread to regional lymph nodes, liver, and lung, despite comparable primary tumor growth and histopathological characteristics across genotypes. Notably, Unc5b-deficient tumors exhibited extensive intratumoral necrosis, reduced Ki67-driven proliferative capacity, and a lack of local tissue invasion, underscoring a critical role for Unc5b in sustaining tumor cell viability, proliferation, and the ability to breach tissue boundaries and colonize distant sites. Orthotopic implantation of Unc5b knockdown in PDAC cells recapitulated these findings. We and others have observed UNC5B expression in both primary and metastatic PDAC tumors yet there appears to be no expression of it in normal pancreatic tissue. An analysis of pancreatic cancer genomes revealed aberrations in axon guidance molecules however this was mostly in the SLIT/ROBO signaling pathway with very few copy number variances found in UNC5B [24]. Most likely UNC5B is regulated epigenetically during tumorigenesis which is consistent with other studies of PDAC metastasis [25]. It is unclear how or when during PDAC tumorigenesis that UNC5B expression is turned on but given that PDAC has been shown to disseminate early during tumorigenesis [26], we speculate that UNC5B may turn on before the stage of invasive PDAC. Together, these results delineate a critical function for UNC5B in orchestrating PDAC invasion and metastatic progression and provide a mechanistic framework linking its expression to disease aggressiveness in vivo and in human patients.

While EMT has been shown to be an important process that facilitates metastasis in multiple solid organ cancers including PDAC, the mechanisms that regulate EMT in PDAC have until now been unclear. Our research provides an important link between Netrin-1/UNC5B signaling with ZEB1 and EMT in PDAC. Moreover, NP137 is an antibody drug that inhibits the Netrin-1/UNC5B interaction and was recently shown in both murine and human gynecologic cancer to inhibit EMT which is consist with our results [27, 28]. Our data identify NP137 as a therapeutic that could potentially inhibit EMT and metastasis through disrupting the UNC5B signaling in PDAC. This has led to a “window of opportunity” clinical trial that is designed to determine if NP137 can inhibit EMT in human pancreatic cancer patients (NCT05546853). This represents an entirely new therapeutic approach to PDAC.

Our mechanistic analyses identify UNC5B as a regulator of EMT and invasion through modulation of the SRC-ZEB1 signaling axis. A similar finding was reported for the regulation of EMT in gastric cancer [29]. It is unclear precisely how UNC5B regulates SRC as it appears to be through both transcriptional and post-translational mechanisms which may be distinct. Elucidating how UNC5B interfaces with SRC signaling may therefore uncover previously unrecognized regulatory layers governing EMT and metastatic competence in PDAC. Nonetheless, SRC signaling has been implicated in the metastatic progression of PDAC [30]. Previous studies using the genetically engineered KPC mouse model demonstrated that pharmacological inhibition of SRC/ABL with Dasatinib markedly suppresses tumor cell migration and invasion in vitro and significantly reduces metastatic burden in vivo. Notably, this anti-metastatic effect occurred without measurable inhibition of primary tumor growth or improvement in overall survival, similar to what we observed with Unc5b deletion in the KPC model [30]. While these findings established SRC signaling as a key driver of PDAC metastasis, clinical translation has proven challenging as a phase II trial investigating the addition of Dasatinib to gemcitabine in patients with locally advanced, non-metastatic PDAC failed to confer a significant survival benefit compared with gemcitabine alone [31]. Dasatinib is a multi-kinase inhibitor, and it is possible that the lack of efficacy could be due to off target effects. Our data place UNC5B as a key upstream regulator of both SRC and ZEB1 in PDAC to regulate both EMT and metastasis and suggest that targeting upstream regulators of SRC rather than SRC itself, may offer a more effective strategy for limiting metastatic progression. UNC5B emerges as a potential central node through which PDAC cells acquire metastatic competency. These insights not only advance our understanding of the molecular mechanisms underpinning PDAC metastasis but also provides a new therapeutic strategy to target it.

## Supplementary information

Supplementary Figures 1

Supplementary Methods 1

## Data Availability

The data supporting the findings of this study are available within the article and its Supplementary Information files. Additional data are available from the corresponding author upon reasonable request for non-commercial research purposes.

## References

[1] Hu ZI, O’Reilly EM. Therapeutic developments in pancreatic cancer. Nat Rev Gastroenterol Hepatol. 2024;21:7–24. 37798442 10.1038/s41575-023-00840-w

[2] Jones RP, Psarelli E-E, Jackson R, Ghaneh P, Halloran CM, Palmer DH, et al. Patterns of recurrence after resection of pancreatic ductal adenocarcinoma: a secondary analysis of the ESPAC-4 randomized adjuvant chemotherapy trial. JAMA Surg. 2019;154:1038–48. 31483448 10.1001/jamasurg.2019.3337 PMC6727687

[3] Rahib L, Smith BD, Aizenberg R, Rosenzweig AB, Fleshman JM, Matrisian LM. Projecting cancer incidence and deaths to 2030: the unexpected burden of thyroid, liver, and pancreas cancers in the United States. Cancer Res. 2014;74:2913–21. 24840647 10.1158/0008-5472.CAN-14-0155

[4] Yang J, Antin P, Berx G, Blanpain C, Brabletz T, Bronner M, et al. Guidelines and definitions for research on epithelial-mesenchymal transition. Nat Rev Mol Cell Biol. 2020;21:341–52. 32300252 10.1038/s41580-020-0237-9 PMC7250738

[5] Greco L, Rubbino F, Laghi L. Epithelial to mesenchymal transition as mechanism of progression of pancreatic cancer: from mice to men. Cancers (Basel). 2022;14:5797. 10.3390/cancers14235797 PMC9735867 36497278

[6] Yang J, Liu Y, Liu S. The role of epithelial-mesenchymal transition and autophagy in pancreatic ductal adenocarcinoma invasion. Cell Death Dis. 2023;14:506. 37550301 10.1038/s41419-023-06032-3 PMC10406904

[7] Zheng X, Carstens JL, Kim J, Scheible M, Kaye J, Sugimoto H, et al. Epithelial-to-mesenchymal transition is dispensable for metastasis but induces chemoresistance in pancreatic cancer. Nature. 2015;527:525–30. 26560028 10.1038/nature16064 PMC4849281

[8] Krebs AM, Mitschke J, Lasierra Losada M, Schmalhofer O, Boerries M, Busch H, et al. The EMT-activator Zeb1 is a key factor for cell plasticity and promotes metastasis in pancreatic cancer. Nat Cell Biol. 2017;19:518–29. 28414315 10.1038/ncb3513

[9] Dudgeon C, Casabianca A, Harris C, Ogier C, Bellina M, Fiore S, et al. Netrin-1 feedforward mechanism promotes pancreatic cancer liver metastasis via hepatic stellate cell activation, retinoid, and ELF3 signaling. Cell Rep. 2023;42:113369. 37922311 10.1016/j.celrep.2023.113369 PMC12270551

[10] Zhu Y, Li Y, Nakagawara A. UNC5 dependence receptor family in human cancer: a controllable double-edged sword. Cancer Lett. 2021;516:28–35. 34077783 10.1016/j.canlet.2021.05.034

[11] Hingorani SR, Wang L, Multani AS, Combs C, Deramaudt TB, Hruban RH, et al. Trp53R172H and KrasG12D cooperate to promote chromosomal instability and widely metastatic pancreatic ductal adenocarcinoma in mice. Cancer Cell. 2005;7:469–83. 15894267 10.1016/j.ccr.2005.04.023

[12] Jackson EL, Willis N, Mercer K, Bronson RT, Crowley D, Montoya R, et al. Analysis of lung tumor initiation and progression using conditional expression of oncogenic K-ras. Genes Dev. 2001;15:3243–8. 11751630 10.1101/gad.943001 PMC312845

[13] Wilson BD, Ii M, Park KW, Suli A, Sorensen LK, Larrieu-Lahargue F, et al. Netrins promote developmental and therapeutic angiogenesis. Science. 2006;313:640–4. 16809490 10.1126/science.1124704 PMC2577078

[14] Olive KP, Tuveson DA, Ruhe ZC, Yin B, Willis NA, Bronson RT, et al. Mutant p53 gain of function in two mouse models of Li-Fraumeni syndrome. Cell. 2004;119:847–60. 15607980 10.1016/j.cell.2004.11.004

[15] Hingorani SR, Petricoin EF, Maitra A, Rajapakse V, King C, Jacobetz MA, et al. Preinvasive and invasive ductal pancreatic cancer and its early detection in the mouse. Cancer Cell. 2003;4:437–50. 14706336 10.1016/s1535-6108(03)00309-x

[16] Stifter SA, Greter M. STOP floxing around: Specificity and leakiness of inducible Cre/loxP systems. Eur J Immunol. 2020;50:338–41. 32125704 10.1002/eji.202048546

[17] Lee JW, Komar CA, Bengsch F, Graham K, Beatty GL. Genetically Engineered Mouse Models of Pancreatic Cancer: The KPC Model (LSL-Kras(G12D/+) ;LSL-Trp53(R172H/+) ;Pdx-1-Cre), Its Variants, and Their Application in Immuno-oncology Drug Discovery. Curr Protoc Pharm. 2016;73:14.39.1–14.39.20. 10.1002/cpph.2 PMC4915217 27248578

[18] Taherian M, Wang H, Wang H. Pancreatic ductal adenocarcinoma: molecular pathology and predictive biomarkers. Cells. 2022;11:3068. 10.3390/cells11193068 PMC9563270 36231030

[19] Mak MP, Tong P, Diao L, Cardnell RJ, Gibbons DL, William WN, et al. A patient-derived, pan-cancer EMT signature identifies global molecular alterations and immune target enrichment following epithelial-to-mesenchymal transition. Clin Cancer Res. 2016;22:609–20. 26420858 10.1158/1078-0432.CCR-15-0876 PMC4737991

[20] Nicolle R, Blum Y, Duconseil P, Vanbrugghe C, Brandone N, Poizat F, et al. Establishment of a pancreatic adenocarcinoma molecular gradient (PAMG) that predicts the clinical outcome of pancreatic cancer. EBioMedicine. 2020;57:102858. 32629389 10.1016/j.ebiom.2020.102858 PMC7334821

[21] Lee J, Li W, Guan KL. SRC-1 mediates UNC-5 signaling in Caenorhabditis elegans. Mol Cell Biol. 2005;25:6485–95. 16024786 10.1128/MCB.25.15.6485-6495.2005 PMC1190325

[22] Raji L, Tetteh A, Amin A role of c-Src in carcinogenesis and drug resistance. Cancers (Basel). 2023;16:32. 10.3390/cancers16010032 PMC10778207 38201459

[23] Dey N, Crosswell HE, De P, Parsons R, Peng Q, Su JD, et al. The protein phosphatase activity of PTEN regulates SRC family kinases and controls glioma migration. Cancer Res. 2008;68:1862–71. 18339867 10.1158/0008-5472.CAN-07-1182

[24] Biankin AV, Waddell N, Kassahn KS, Gingras M-C, Muthuswamy LB, Johns AL, et al. Pancreatic cancer genomes reveal aberrations in axon guidance pathway genes. Nature. 2012;491:399–405. 23103869 10.1038/nature11547 PMC3530898

[25] McDonald OG, Li X, Saunders T, Tryggvadottir R, Mentch SJ, Warmoes MO, et al. Epigenomic reprogramming during pancreatic cancer progression links anabolic glucose metabolism to distant metastasis. Nat Genet. 2017;49:367–76. 28092686 10.1038/ng.3753 PMC5695682

[26] Rhim AD, Mirek ET, Aiello NM, Maitra A, Bailey JM, McAllister F, et al. EMT and dissemination precede pancreatic tumor formation. Cell. 2012;148:349–61. 22265420 10.1016/j.cell.2011.11.025 PMC3266542

[27] Cassier PA, Navaridas R, Bellina M, Rama N, Ducarouge B, Hernandez-Vargas H, et al. Netrin-1 blockade inhibits tumour growth and EMT features in endometrial cancer. Nature. 2023;620:409–16. 37532934 10.1038/s41586-023-06367-z PMC10412451

[28] Lengrand J, Pastushenko I, Vanuytven S, Song Y, Venet D, Sarate RM, et al. Pharmacological targeting of netrin-1 inhibits EMT in cancer. Nature. 2023;620:402–8. 37532929 10.1038/s41586-023-06372-2 PMC7615210

[29] Zhao L, Li X, Song N, Li A, Hou K, Qu X, et al. Src promotes EGF-induced epithelial-to-mesenchymal transition and migration in gastric cancer cells by upregulating ZEB1 and ZEB2 through AKT. Cell Biol Int. 2018;42:294–302. 29052277 10.1002/cbin.10894

[30] Morton JP, Karim SA, Graham K, Timpson P, Jamieson N, Athineos D, et al. Dasatinib inhibits the development of metastases in a mouse model of pancreatic ductal adenocarcinoma. Gastroenterology. 2010;139:292–303. 20303350 10.1053/j.gastro.2010.03.034

[31] Evans TRJ, Van Cutsem E, Moore MJ, Bazin IS, Rosemurgy A, Bodoky G, et al. Phase 2 placebo-controlled, double-blind trial of dasatinib added to gemcitabine for patients with locally-advanced pancreatic cancer. Ann Oncol. 2017;28:354–61. 27998964 10.1093/annonc/mdw607

