# Supplementary material for: UNC5B regulates epithelial-to-mesenchymal transition through a SRC-ZEB1 signaling axis to facilitate pancreatic cancer metastasis
Source: Oncogene. 2026 Aug 1;45(37):3949–60. doi: 10.1038/s41388-026-03908-4 (PMC13549943; doi:10.1038/s41388-026-03908-4)
Supplement: Supplementary file 1 [file 41388_2026_3908_MOESM1_ESM.docx]

**Figure S1**. **UNC5B is highly expressed by PDAC cells, and its expression promotes metastasis in pancreatic cancer. A**) In situ hybridization images showing Unc5b expression across KPCU groups. KPCU^+/+^ tumors exhibit high levels of Unc5b expression, whereas KPCU^-/-^ tumors show a complete loss of Unc5b signal. **B**) In situ hybridization analysis of primary pancreatic tumors from KPCU^-/-^ mice that developed overt liver metastases. Despite Cre-LoxP introduction, these tumors showed persistent Unc5b expression, indicating incomplete recombination in the lesions that subsequently metastasized. **C**) Immunocytochemistry (ICC) analysis of Ki67 in Met38 pancreatic cancer cells demonstrated that UNC5B knockdown (KD) resulted in reduced Ki67 expression compared to wild-type controls. **D**) schematic illustration of pancreas orthotopic models. Western Blot analysis confirms Unc5b KD in murine Met38 cell line. In an orthotopic pancreatic cancer model, stable Unc5b KD in murine PDAC cells (Met38 line) resulted in a marked reduction in metastatic burden. Mice (n=12/group) were injected orthotopically with either Unc5b wild-type or Unc5b KD cells, followed by primary tumor resection at almost 2 weeks post-injection and followed up. Subsequent analysis of distant organs showed that all mice in the Unc5b wild-type group developed overt metastases. In contrast, mice in the Unc5b KD group displayed no detectable metastases (*****P* < 0.0001, unpaired two-tailed *t*-test). **E)** UNC5B expression landscape across 176 human pancreatic cancer samples from the TCGA cohort, illustrating the distribution and variability of UNC5B transcription in patient tumors. Blue dots represent alive individual (n=92) and red dots indicates dead individual (n=84); on the right, Box-and-whisker plot showing UNC5B expression levels in human PDAC tumors with average pTPM of 20.2. **F**) RNA-seq data from a cohort of 289 PDAC patients (GSE205154) demonstrated that UNC5B expression is elevated in both primary and metastatic tumor cells. Statistical analysis was performed using unpaired *t*-tests between groups. **G**) Analysis of 45 matched pairs of human PDAC tumors and adjacent non-tumor pancreatic tissues (GDS4336) revealed significantly higher UNC5B expression in PDAC tumor cells compared with normal pancreas (non-tumor tissues). In the tissue-type annotation, dark green indicates PDAC tumor tissue, whereas light green represents adjacent non-tumor pancreatic tissue. In disease state annotation, dark, medium, and light purple represents cancer death NA, 1, and 0, respectively. **H**) representative Image of In situ hybridization showing robust Unc5b expression in a primary pancreatic tumor from a KPCU^+/+^ mouse (n=10), with no detectable Unc5b signal in normal mouse pancreas (n=4).

**Supp Figure S2**. **UNC5B Drives Mesenchymal Transition and Regulates EMT in PDAC. A)** MAK EMT scores, based on a 77-gene signature, were calculated from RNA-seq data of primary tumors from KPCU^+/+^ (wild-type), KPCU^+/-^, and KPCU^-/-^ mice. KPCU^-/-^ tumors exhibited a lower EMT scores compared to wild-type tumors (P=0.0976), suggesting a shift toward a more epithelial-like, less aggressive phenotype. **B**), Representative double immunohistochemistry (IHC) staining for E-cadherin (epithelial marker) and Vimentin (mesenchymal marker) in primary tumors from KPCU mice (n=8 per group). KPCU^-/-^ tumors exhibited strong E-cadherin expression with minimal Vimentin staining, consistent with an epithelial phenotype. In contrast, KPCU^+/+^ and KPCU^+/-^ tumors displayed heterogeneous staining patterns, comprising both E-cadherin-positive and Vimentin-positive cell populations, with KPCU^+/+^ tumors showing a predominance of Vimentin-positive cells indicative of enhanced mesenchymal features. **C**) Western blot analysis confirms UNC5B overexpression and depletion in KP4 pancreatic cancer cells. UNC5B overexpression reduced E-cadherin expression, whereas UNC5B depletion increased E-cadherin levels. Immunocytochemistry and Western blot analyses demonstrated a marked upregulation of vimentin expression in KP4 cells overexpressing UNC5B compared with empty vector-transduced controls, indicating an enhanced acquisition of mesenchymal characteristics upon UNC5B overexpression. **D**) Western blot analyses indicate moderate UNC5B knockdown in human MIA PaCa-2 cells. Widefield light microscopy shows UNC5B KD led to epithelial-like morphology in MIA PaCa-2 cells. CRISPR activation-mediated endogenous overexpression of UNC5B enhanced the mesenchymal phenotype of MIA PaCa-2 cells, as indicated by EMT and PAMG scoring systems. **E**) Conversely, UNC5B knockdown in murine Met38 cells reduced mesenchymal scoring (higher PAMG score), indicating an epithelial shift. Western blot analyses indicate that Unc5b knockdown in murine Met38 cell line reduces the vimetin expression **F**) Gene set enrichment and pathway analysis were performed to identify biological processes and signaling pathways modulated by receptor expression across the indicated comparisons, UNC5B overexpression (UNC5B OE) and UNC5B depletion using Degron construct (UNC5B DP). The bubble plot depicts significantly enriched Gene Ontology (GO) terms and MSigDB/KEGG pathways, ranked by combined enrichment score. Bubble size corresponds to the adjusted *p*-value, and colors indicate the comparison groups. Statistical comparisons between groups were conducted using unpaired two-tailed Student’s t-tests and p-values < 0.05 were considered statistically significant.

**Figure S3**. **UNC5B Facilitates Epithelial-Mesenchymal Transition Through SRC/ZEB1 Regulation. A)** UNC5B KD in MIA PaCa2 cells led to a significant decrease in ZEB1 levels, as measured by western blot, with no significant changes observed in other canonical EMT-inducing transcription factors (e.g., SNAIL1, SLUG, TWIST1). **B**) Exogenous UNC5B overexpression significantly increased ZEB1 level, without affecting the expression of other EMT-inducing transcription factors. ZEB1 protein levels were further validated by immunocytochemistry (ICC) in the same cells, confirming increased nuclear ZEB1 expression upon UNC5B overexpression. **C**) Acute degradation of UNC5B reduces ZEB1 expression in human pancreatic cancer cells. UNC5B expression was modulated using a protein degrader (Degron) system in human BxPC-3 cells stably overexpressing UNC5B fused to the FKBP^12^F36V^ degron tag. Acute degradation of UNC5B upon dTAG^V^-1 treatment resulted in a no significant reduction of canonical EMT-inducing transcription factors, including SNAIL1 and SLUG, with undetectable level of TWIST1 remained unchanged. Gene expression was assessed by qPCR. **D**) Western blot analysis shows UNC5B OE elevated the ZEB1 expression and acute depletion of UNC5B using dTAG^V^-1 compound reverted ZEB1 level to the baseline. Statistical significance was determined using an unpaired *t*-test. *p* < 0.05 was considered significant. **E**) Western blot analysis confirms UNC5B overexpression in human AsPC-1 and PANC-1 pancreatic cancer cell lines. **F**) Western Blot analysis showing the modulation of PTEN in human BxPC3 pancreatic cell line with UNC5B overexpression and depletion models.

**Supplementary Figure 1**

**B**

**A**

**Liver Mets**

**KPCU-/-**

**KPCU-/-**

**KPCU+/-**

**KPCU+/+**

**C**

**UNC5B KD**

**Wild Met38**

**Merged**

**Ki67**

**DAPI**,

Unc5b KD

Beta-Tubulin

Unc5b

Wild Met 38

130 kDa

**D**

55 kDa

**F**

**E**

**N=176**

**UNC5B expression**

**G**

**Representative colors**

**Tissue type**

**Disease State**

**H**

Mouse Normal Pancreas

Mouse PDAC Primary Tumor

**Supplemental Figure 2**

**A**

**B**

**40X**

**40X**

**40X**

**E-cadherin, Vimentin**

KPCU-/-

KPCU+/-

KPCU+/+

**10X**

**10X**

**10X**

**C**

55kDa

Beta tubulin

E-Cadherin

135 kDa

150kDa

120kDa

Exo UNC5B

UNC5B OE/DP

KP4 UNC5B OE

Endo UNC5B

Endo UNC5B

Exo UNC5B

UNC5B OE

Beta tubulin

120kDa

55kDa

150kDa

KP4 EV

KP4 EV

UNC5B OE

Vimentin

Beta Tubulin

57 KDa

55 KDa

**Vimentin**

**Merged**

**DAPI**

KP4 EV

UNC5B OE

**Merged**

**DAPI**

**Vimentin**

**D**

MIA-PACA2 EV

UNC5B

55 KDa

UNC5B KD

120 KDa

Beta Tubulin

MIA PACA-2 EV

UNC5B KD

Wild Met 38

Unc5b KD

**E**

57 KDa

55 KDa

Vimentin

Beta tubulin

**F**

KEGG and MSigDB hallmarks UP

KEGG and MSigDB hallmarks Down

**Supplemental Figure 3**

**A**

55 kDa

200 kDa

ZEB1

Beta Tubulin

29 kDa

26 kDa

Wild MIA-PACA

UNC5B KD

30 kDa

SNAIL

SLUG

TWIST1

**B**

**C**

KP4 EV

UNC5B OE

**Merged**

**DAPI**

**ZEB1**

**Merged**

**DAPI**

UNC5B OE

Wild KP4

SNAIL

ZEB1

29 kDa

200 kDa

TWIST1

30 kDa

SLUG

26 kDa

**ZEB1**

Beta Tubulin

55 kDa

**D**

KP4 EV

UNC5B OE/ DP

UNC5B OE

200 kDa

ZEB1

55 kDa

Beta Tubulin

**E**

UNC5B OE

Wild PANC1

UNC5B OE

Wild ASPC2

120kDa

55kDa

55kDa

120kDa

Beta Tubulin

UNC5B

**F**

Wild BXPC3

UNC5B OE

UNC5B OE/DP

PTEN

Beta Tubulin

55kDa

54kDa
