# Supplementary material for: UNC5B regulates epithelial-to-mesenchymal transition through a SRC-ZEB1 signaling axis to facilitate pancreatic cancer metastasis
Source: Oncogene. 2026 Aug 1;45(37):3949–60. doi: 10.1038/s41388-026-03908-4 (PMC13549943; doi:10.1038/s41388-026-03908-4)
Supplement: Supplementary file 2 [file 41388_2026_3908_MOESM2_ESM.docx]

**Material and Methods**

**Mice**

The *Pdx1-Cre* transgene (Pdx1-cre), the conditional *Kras^LSL.G12D^*, and *Tp53^LSL.R172H^* alleles and the KPC mouse model have been described previously and were maintained on a C57BL/6 genetic background (1-4).The generation of Unc5b knockout allele (Unc5b^flox/flox^) is described elsewhere (5). Briefly, to generate the Unc5b conditional knockout line, a *Unc5b* targeting construct was engineered through homologous recombination to produce the *Unc5b^Neo^* allele. The neomycin resistance cassette was flanked by FRT sites, and Cre recombinase (*Hprt-Cre*) was used to excise exons 4-13, resulting in the *Unc5b*-deleted (*Unc5b−*) allele. Successful germline transmission of this model was confirmed by Southern blot and PCR analysis of offspring derived from chimeric founders. To generate the KPCU mouse model, Unc5b^flox/flox^ mice were crossed with *Pdx1-Cre; Kras^LSL.G12D^*; and *Tp53^LSL.R172H^* (KPC) mice. The resulting progeny (*Pdx1-Cre; Kras^LSL.G12D^*; and *Tp53^LSL.R172H^;* *Unc5b^+/+^*, *^+/-^*, and *^-/-^*) were designated as KPCU^+/+^, KPCU^+/-^, and KPCU^-/-^, respectively. KPCU mice were monitored weekly by abdominal palpation for tumor onset and progression and subjected to ultrasound imaging to confirm pancreatic tumor development. Once the primary tumor reached the maximum tolerated size (approximately 1 cm in diameter), mice were euthanized, and comprehensive necropsies were performed to assess macroscopic evidence of metastatic disease. Primary tumors, lungs, liver, and spleen were collected for microscopic evaluation of metastasis. Harvested primary tumors were divided for subsequent RNA extraction, generation of single-cell suspensions for cell line establishment, and histopathological analyses. Remaining tumor tissues and organs were fixed in 10% neutral buffered formalin (VWR), processed for paraffin embedding, and sectioned for hematoxylin and eosin (H&E) staining and immunohistochemical evaluation by a board-certified pathologist.

All animal studies were conducted in accordance with institutional and national guidelines for the care and use of laboratory animals and were approved by the Institutional Animal Care and Use Committee (IACUC; protocol no. 2020-008E) at the University of Rochester University Committee on Animal Resources (UCAR). Mice were maintained under specific pathogen-free (SPF) conditions in a temperature- and humidity-controlled facility with a 12-hour light/dark cycle and ad libitum access to standard chow and water. Both male and female mice were included in experiments unless otherwise indicated. Health monitoring and genotyping were performed regularly to ensure colony integrity. Mice exhibiting signs of distress, tumor burden exceeding institutional limits, or rapid weight loss (>15%) were humanely euthanized in accordance with IACUC-approved endpoints. FVB mice were purchased from The Jackson Laboratory (Bar Harbor, ME), or bred in-house following established vivarium approved protocols. Young mice (6-8 weeks old) were used for experiment. Mice were monitored daily for survival endpoints.

**Cell lines**

Human pancreatic cancer cell lines, including BxPC-3, MIA PaCa-2, SW1990, AsPC-1, and PANC-1, were obtained from ATCC and authenticated by short tandem repeat (STR) profiling. KP4 cell line was obtained from JCRB cell bank. The human embryonic kidney cell line 293T was also purchased from ATCC.  Met38 is a mouse pancreatic cancer cell line derived from the *p48*^cre^; *Kras*^LSL_G12D^; p16^−/−^/*p19*^−/−^ mouse model. A knockdown of Unc5b was generated in the Met38 cell line by CRISPR-Cas9. All cell lines, except Met38 cell line, were routinely tested and confirmed negative for mycoplasma contamination using the MycoFluor™ Mycoplasma Detection Kit (Invitrogen). Met38 cell line was treated with plasmocin to control the Mycoplasma contamination (InvivoGen). Cells were maintained in complete DMEM (SW1990, MIA PaCa-2, PANC-1, Met38, and 293T) or RPMI (BxPC-3, KP4, and AsPC-1) medium supplemented with 10% fetal bovine serum, 1% penicillin/streptomycin, and L-glutamine under standard culture conditions.

**Orthotopic implantation of pancreatic tumor cells**

Mice were anesthetized with 2-3% isoflurane and placed on a heated surgical platform to maintain body temperature. The abdominal area was shaved and disinfected with alternating swabs of povidone-iodine and 70% ethanol before incision.

Orthotopic pancreatic tumors were generated by injecting either 5000 Met38 cells or Unc5b KD Met38 cells suspended in a 1:1 mixture of Matrigel Matrix (Corning) and complete DMEM (10 µL total volume) into the pancreatic tail using a Hamilton microliter syringe fitted with a 33-gauge needle. A clamp was placed downstream of pancreas head to preserve some healthy pancreas in mice. For distal pancreatectomy with splenectomy, a midline laparotomy was performed to expose the pancreas and spleen. The spleen and primary pancreatic tumor were removed after 17 days post implantation.

Buprenorphine (0.05-0.1 mg/kg, s.c.) and bupivacaine (2 mg/kg, local infiltration) were administered for perioperative analgesia. Following surgery, mice were monitored daily for body weight, grooming, surgical site bleeding, and behavior, and recovered under a heating lamp until ambulatory. Humane endpoints were defined as >20% body weight loss, severe lethargy, or abdominal distension; affected animals were humanely euthanized via CO_2_ asphyxiation followed by cervical dislocation.

**Histology, histopathology and immunohistochemistry**

Primary tumors, Lungs and livers were fixed in 10% neutral buffered formalin (VWR) for 24-48 hours, followed by routine processing and paraffin embedding. Paraffin blocks were sectioned at 5 µm thickness, mounted on glass slides. Sections were deparaffinized in Histoclear (National Diagnostics) for 5 minutes (three changes) and rehydrated through a graded ethanol series (2 × 100%, 2 × 95%, 1 × 70%, 1 × 50%) followed by rinsing in double-distilled water (2×). Antigen retrieval was performed by boiling slides in Tris-Based Antigen Unmasking Solution (Vector Laboratories; cat. #H-3301-250) for 15 minutes, then cooling to room temperature. Slides were treated with Peroxidase suppressor (Thermo Scientific; cat. #35000) for 10 minutes, followed by three washes in TBST buffer (1× PBS with 0.1% Tween-20). Endogenous biotin activity was quenched using a Streptavidin/Biotin Blocking Kit (Vector Laboratories; cat. #SP-2002), with each step performed for 30 minutes at room temperature. Sections were then blocked with 10% normal serum (of the host species of the secondary antibody) for 30 minutes and incubated overnight at 4 °C with primary antibodies (Zeb1; cell signaling technology; cat. #70512S) diluted in blocking buffer. After washing (3× in TBST), sections were incubated with biotinylated Horse Anti-rabbit HRP-conjugated secondary antibodies (Vector Laboratories; cat. #BA-1100) for 1 hour at room temperature. Signal amplification was achieved using the VECTASTAIN ABC-HRP Kit (Vector Laboratories; cat. #PK-6100), and antigen localization was visualized using the ImmPACT DAB Peroxidase Substrate Kit (Vector Laboratories; cat. #SK-4105). Slides were counterstained with hematoxylin, dehydrated through graded ethanol, cleared in xylene, and mounted with Permount Mounting Medium. Images were acquired and quantified for DAB-positive staining using the Keyence BZ-X710 fluorescent microscope with standardized threshold and segmentation parameters applied across all samples.

Histopathological examination was performed by a board-certified veterinary pathologist at the University of Rochester Medical Center in a blinded manner with respect to genotype and treatment group. Evaluations included tumor differentiation, necrosis, lymph node involvement, smooth muscle invasion, and intestinal wall invasion. The presence of metastatic lesions in distant organs (liver and lungs) was confirmed microscopically. Representative images were acquired using a Keyence BZ-X710 fluorescent microscope.

**Immunocytochemistry (ICC)**

Human KP4 cells stably overexpressing the UNC5B receptor were seeded at a density of 50,000 cells per well on sterile glass coverslips placed in 12-well plates. After 48 hours, cells were fixed with 4% paraformaldehyde (PFA) at room temperature for 10 minutes and permeabilized with 0.25% Triton X-100 for 10 minutes. Following three washes with PBS, cells were incubated overnight at 4 °C with primary antibodies against Zeb1 or vimentin (Cell Signaling Technology; cat. #70512S, #5741S). After washing, cells were incubated for 1 hour at room temperature with Alexa Fluor 488-conjugated anti-rabbit secondary antibody (Invitrogen; cat. #A11008). Coverslips were mounted using VECTASHIELD Antifade Mounting Medium with DAPI (Vector Laboratories) to visualize nuclei. Images were acquired using a Keyence BZ-X710 fluorescence microscope under identical exposure settings across all experimental groups.

**Immunofluorescence (IF)**

Five-micrometer sections were cut from formalin-fixed, paraffin-embedded (FFPE) blocks. Sections were deparaffinized in Histoclear (National Diagnostics) for 5 minutes, with three changes, and rehydrated through a graded ethanol series (2 × 100%, 2 × 95%, 1 × 70%, 1 × 50%), followed by two rinses in double-distilled water. Antigen retrieval was performed by incubating slides in citrate buffer at 100 °C for 15 minutes. Slides were blocked for 1 hour at room temperature in phosphate-buffered saline with 0.05% Tween-20 (PBST) and 10% bovine serum albumin (BSA). Sections were then incubated overnight at 4 °C with primary anti-vimentin antibody conjugated to Alexa Fluor 647 (Cell signaling technology cat. #9856S) diluted 1:250 in blocking buffer. The following day, slides were washed twice in PBST 0.05% and once in 1× PBS for 3 minutes each, blotted dry, and counterstained with DAPI (10 µg/mL in 1× PBS). Finally, coverslips were applied, and images were acquired using a Keyence BZ-X710 fluorescence microscope.

**In situ Hybridization (RNAscope) Technique**

Formalin-fixed, paraffin-embedded (FFPE) tissue sections (5 µm) were deparaffinized in Histoclear (National Diagnostics) for 5 minutes with three changes and rehydrated through 100% ethanol for 1 minute. Slides were then air-dried for 5 minutes. Endogenous peroxidase activity was quenched by applying 5-8 drops of RNAscope hydrogen peroxidase reagent (ACD; cat. #322381) to each slide for 10 minutes at room temperature, followed by three washes with distilled water.

Slides were subjected to antigen retrieval by immersion in boiling 1× RNAscope antigen retrieval solution (ACD; cat# 322001) for 15 minutes. After cooling to room temperature, slides were washed three times with distilled water, transferred into 100% ethanol, and air-dried. A hydrophobic barrier was drawn around the tissue sections to confine reagents. Protease treatment was performed using RNAscope protease solution (ACD; cat# 322381) at 40 °C for 30 minutes, followed by three washes in distilled water.

Hybridization was carried out using the target mouse Unc5b probe (ACD; cat. #482481) and negative probe (ACD; cat. #310043) as a control, applied to fully cover the tissue sections, and incubated at 40°C in a humidified chamber for 2 hours. Slides were then washed with 1× RNAscope wash buffer (ACD; cat. #310091). Signal amplification was performed sequentially using a series of amplification reagents (AMP1-AMP6) (ACD; cat. #322310), each incubated for 15-30 minutes at 40°C according to the manufacturer’s protocol, with washes in wash buffer after each step.

Visualization of the signal was achieved by applying an equal volume mixture of DAB-A and DAB-B (ACD; cat. #322310) for up to 10 minutes, followed by immersion in distilled water. Slides were counterstained with hematoxylin for 2 minutes, briefly rinsed, and dipped 2-3 times in 0.02% ammonia water. Sections were dehydrated through a graded ethanol series (70%, 90%, 95%, 100%), cleared in xylene, and mounted with a permanent mounting medium. Slides were analyzed and scored according to the RNAscope manufacturer’s guidelines.

**Protein Degrader (Degron) Development**

Degron construct was developed as described previously(6). Briefly, to generate the pDONR construct, full-length human UNC5B PCR products were cloned into the pDONR221 plasmid (Invitrogen) using the Gateway BP Clonase II enzyme mix (Invitrogen, Cat# 11789020) according to the manufacturer’s protocol. The purified PCR products and pDONR221 vector were incubated with BP Clonase II enzyme. Following the reaction, 2 µL of Proteinase K (2 µg/µL) was added and incubated at 37 °C for 10 minutes to terminate the reaction. The resulting mixture was transformed into Stbl3 competent E. coli cells, and colonies were selected on LB agar plates. Correct insertion of UNC5B into the pDONR221 vector was verified by DNA sequencing. Plasmid DNA was extracted using the QIAGEN Plasmid Mini Kit.

Subsequently, an LR recombination reaction was performed to transfer the UNC5B coding sequence from pDONR221 into the pLEX_305-C-dTAG expression vector (a gift from James Bradner & Behnam Nabet; Addgene #91798). The pDONR221-UNC5B plasmid and pLEX_305-N-dTAG vector were combined in the presence of Gateway LR Clonase II enzyme according to the manufacturer’s instructions Invitrogen, Cat. #11791020). The LR reaction mixture was then transformed into NEB 10-beta competent E. coli, and transformants were grown on selective media. Correct recombinant clones were confirmed by sequencing, and plasmid DNA was extracted for downstream applications. This approach allowed for the generation of a full-length human UNC5B degron construct suitable for inducible protein degradation studies in mammalian cells.

UNC5B depletion using the dTAG system**:** For UNC5B depletion experiments, stable PDAC cell lines expressing the UNC5B-Degron construct were plated in 6-well plates or 10cm tissue culture dishes and maintained at 37 °C in a humidified incubator with 5% CO_2_. The dTAG^V^-1 compound (Tocris, Cat. #6914) was supplied as a 5 mg solid and dissolved in DMSO to prepare a 10 mM stock solution, which was stored at −20 °C. The negative control compound, dTAG-13-Neg (Tocris, Cat. #6916), was prepared similarly. For treatments, cells were exposed to dTAG^V^-1 at a final concentration of 750 nM, while control cells received an equivalent volume of or dTAG-13-Neg. Following compound addition, cells were incubated for 48 hours, after which they were collected for downstream analyses including RNA extraction, protein quantification, and functional assays.

**Vector development for Gene Overexpression or Knockdown, and lentivirus production**

Lentiviral particles for CRISPR interference (CRISPRi)-mediated knockdown of human *Zeb1* and *SRC* were generated by subcloning 20-nucleotide guide sequences targeting the promoter or transcription start site (TSS) region of each gene into the vector pLV-hU6-sgRNA hUBC-dCas9-KRAB-T2A-Puro (a gift from Charles Gersbach; Addgene plasmid #71236). The sgRNA sequences used were: *ZEB1*, 5′-GGC GCA ATA ACG GTG AGT GG-3′; and *SRC*, 5′-GGG CAG ACG GAC GCA CGG GA-3′. In certain experiments, an alternative backbone, pLV-hU6-sgRNA hUBC-dCas9-KRAB-T2A-Bsd containing a Blasticidin resistance gene (a gift from Stephano Mello), was used instead of the puromycin-selectable version. To generate UNC5B KD cell lines, we used the lentiviral vector lentiCRISPR v2 (a gift from Feng Zhang; Addgene plasmid #52961), by cloning sgRNA (5′-TAG-ACC-TTG-ACC-TTA AGG-CT 3′) targeting human UNC5B between Exon 9-10.

Overexpression oof UNC5B was achieved using two complementary approaches. (1) CRISPR activation (CRISPRa) system: A lentiviral-based CRISPR activation system was established using lenti dCas9-VP64-Blast and lenti sgRNA (MS2)-Puro vectors (gifts from Feng Zhang; Addgene plasmids #61425 and #73795). A single-guide RNA (sgRNA) targeting the promoter region of human UNC5B (5′-GGG GCC TGG GCG ACC GAG GG-3′) was cloned into the lenti sgRNA (MS2)-Puro plasmid according to the Zhang lab protocol. Lentiviral particles were generated and used to transduce target pancreatic cancer cell lines with both CRISPRa components in separately. Following antibiotic selection with Blasticidin (15 µg/mL) and puromycin (5 µg/mL), stable lines expressing dCas9-VP64 and UNC5B-targeting sgRNA were established. Successful UNC5B activation was verified by quantitative RT-PCR and western blotting. (2) Degron-based UNC5B overexpression construct: To enable inducible overexpression and depletion studies, a Degron construct encoding the full-length UNC5B cDNA was generated using the pLEX_305-N-dTAG vector backbone (a gift from James Bradner & Behnam Nabet; Addgene plasmid #91797). This system allows expression of UNC5B fused to a mutated FKABP degron domain, enabling selective degradation of UNC5B protein upon treatment with the dTAG^V^-1 compound. This design was employed to investigate both the functional overexpression and acute depletion effects of UNC5B in pancreatic cancer models.

All sgRNAs cloning was performed using the In-Fusion Snap Assembly Master Mix (Takara Bio, Cat. #638948) following the manufacturer’s instructions. Briefly, destination vectors were linearized by restriction enzyme digestion supplied by (New England Biolabs), and the gene inserts were either PCR-amplified or generated by oligonucleotide annealing using primers containing 15 bp overlaps homologous to the vector ends. Purified vector and insert were mixed at a 1:1 molar ratio with the In-Fusion Snap Assembly reaction mix and incubated at 50 °C for 15 min to enable seamless recombination. The assembled constructs were transformed into Stbl3 competent cells, and positive clones were verified by DNA sequencing.

Lentiviral particles were produced in HEK293T packaging cells by co-transfection with the transfer vector and helper plasmids pMD2.G and psPAX2 (gifts from Didier Trono; Addgene plasmids #12259 and #12260) using Lipofectamine 3000 (Invitrogen; cat. #L3000008) according to the manufacturer’s protocol. After 6 hours, the transfection medium was replaced with fresh complete DMEM. Viral supernatants were collected at 24- and 48-hours post-transfection, filtered through a 0.45 µm PVDF filter, and used immediately for infection. Target pancreatic cancer cell lines were transduced with viral supernatant in the presence of 5µg/mL polybrene (Santa Cruz Biotechnology; cat. #sc-134220) for 8 hours per day across two consecutive days to enhance transduction efficiency. At 48 hours post-transduction, antibiotic selection was initiated using puromycin (5µg/mL) or blasticidin (15 µg/mL) for 5-7 days to establish stable Degron, CRISPRi, or CRISPR activation cell populations. Knockdown or overexpression efficiency was verified by quantitative RT-PCR and western blotting to confirm depletion of the target gene.

**Matrigel Transwell Invasion Assay**

Cell invasion was assessed using Corning BioCoat Matrigel Invasion Chambers (Corning; cat. #354480) following the manufacturer’s instructions. Briefly, chambers were hydrated with base medium for 2 hours at 37°C. PDAC cells were harvested and counted using trypan blue exclusion to ensure viability, and 20,000-40,000 cells were seeded per insert depending on the cell type and experimental purpose. Medium containing 20% FBS was used as a chemoattractant in the lower chamber. After incubation for 22-24 hours at 37°C in a humidified incubator with 5% CO_2_, non-invading cells were removed from the upper membrane surface, and invaded cells on the lower surface were stained using Kwik-Diff Stain (Epredia; cat. #9990700) or Hematoxylin (Vector laboratories; cat. #H-3401-500). After drying, invaded cells were imaged using a Keyence BZ-X710 fluorescence microscope, and invasion was quantified as the average number of invaded cells per field at 10× magnification from three random fields per insert. All experiments were performed in three biological replicates.

**Wound Healing Assay**

Cell migration was evaluated using a wound healing assay with silicone culture inserts that allow formation of a reproducible, cell-free gap (IBIDI; cat. #81176**)**. Inserts were placed in 35-mm culture dishes and cells were seeded into the two compartments at equal density to generate a confluent monolayer. Cells were allowed to adhere and reach confluence overnight under standard culture conditions. Once confluence was achieved, the inserts were carefully removed to create a defined gap between the two cell populations. Wells were gently washed with phosphate-buffered saline or media to remove detached cells, and fresh culture medium was added. Images of the wound area were acquired immediately after insert removal (0 h) and at 12h time points using phase-contrast microscopy at fixed positions. Wound closure was expressed as the percentage reduction in gap area over time. All experiments were performed in at least three independent biological replicates.

**Drug treatment and cell viability assay**

Pancreatic ductal adenocarcinoma cells were seeded at 5,000 cells per well in 96-well plates and allowed to adhere overnight. Compounds were prepared as stock solutions in DMSO and freshly diluted to the indicated concentrations in complete culture medium immediately prior to treatment. The following compounds were used: Dasatinib (MedChemExpress, #HY-10181). Cells were treated with serial dilutions of each drug for 72 h.

Cell viability was assessed using the Cell Counting Kit-8 (CCK-8; Dojindo, cat. #CK04-13) according to the manufacturer’s instructions. Briefly, 10 µL of CCK-8 reagent was added to each well and incubated for 1-2 h at 37 °C, after which absorbance was measured at 450 nm using a microplate reader. Absorbance values were background-subtracted using blank wells and normalized to the mean of vehicle (DMSO)-treated controls. Half-maximal inhibitory concentration (IC_50_) values were calculated by nonlinear regression of dose-response curves using GraphPad Prism (version 10, GraphPad Software). All treatments were performed in triplicate technical replicates and repeated in at least three independent biological experiments.

**Bulk RNA-seq and library preparation**

Total RNA was extracted from PDAC cells using the RNeasy Mini Kit (Qiagen, Cat. #74106) following the manufacturer’s instructions, including on-column DNase digestion to remove genomic DNA contamination. RNA quality and integrity were assessed using a NanoDrop spectrophotometer (ThermoFisher Scientific), and samples with RIN ≥ 8 were used for library preparation. For demultiplexing, raw reads generated from Illumina base calls were processed using bcl2fastq (v2.20.0). Quality filtering and adapter trimming were performed with FastP (v0.23.1).

**Quantitative PCR (qPCR) Analysis of Cell Lines**

Total RNA was isolated from cultured cells as described earlier. RNA integrity and concentration were assessed using a NanoDrop spectrophotometer (ThermoFisher Scientific). Complementary DNA (cDNA) was synthesized from 500 ng to 1 µg of total RNA using the TaqMan Reverse Transcription Reagents (Applied Biosystems cat. #N8080234) according to the manufacturer’s instructions, with random hexamers and MultiScribe Reverse Transcriptase.

Gene expression was quantified using commercial TaqMan Gene Expression Assays (Thermo Scientific). qPCR reactions were performed in triplicate for each sample on a 7500 Real-Time PCR System (Applied Biosystems). Reactions contained (20 µL), 1× TaqMan Universal PCR Master Mix (No AmpErase UNG), and 1-2 µL of cDNA template. Thermal cycling conditions were as follows: initial denaturation at 95 °C for 10 min, followed by 40 cycles of 95 °C for 15 s and 60 °C for 1 min. Relative gene expression levels were calculated using the comparative Ct (ΔΔCt) method, with normalization to human or mouse β-actin as an internal control. Data are presented as mean ± s.d. from at least three independent biological replicates.

**Western Blot Analysis**

Cells were harvested and lysed in RIPA buffer supplemented with phosphatase inhibitor cocktail and PMSF (both from Cell Signaling Technology, CST). Protein concentration was quantified using the Pierce BCA Protein Assay Kit (Thermo Scientific; cat. #23223) according to the manufacturer’s instructions. Equal amounts of total protein were mixed with 4× Laemmli sample buffer containing β-mercaptoethanol (1:100), resolved on Mini-PROTEAN TGX precast gels (Bio-Rad) in 1× TGS running buffer, and transferred to PVDF membranes using semi-dry transfer. Membranes were blocked in 5% non-fat milk in TBST for 1h at room temperature and incubated overnight at 4 °C with the following primary antibodies (all from Cell Signaling Technology): ZEB1 (70512), SRC (2109), phospho-SRC (Tyr530, 2105), PTEN (9188), SLUG (9585), SNAIL (3879), TWIST1 (90445), Vimentin (5741), E-cadherin (24E10), and UNC5B (13851). UNC5B (Bioss #BS-11492R). β-Tubulin (ab6046, Abcam) was used as a loading control. After three washes in TBST, membranes were incubated for 1h at room temperature with the appropriate HRP-conjugated secondary antibodies diluted in blocking buffer. Blots were developed using either Western Lightning Plus-ECL (Revvity Health Sciences) or SuperSignal West Femto Maximum Sensitivity Substrate (Thermo Fisher Scientific). Chemiluminescent signals were captured with a ChemiDoc MP Imaging System (Bio-Rad) and quantified using Image Lab software.

**RNA-seq Analysis**

PDAC cell lines and Tissues were profiled using standard RNA-seq completed by Azenta Life Sciences (formally known as Genewiz, South Plainfield, NJ). After quality check of the reads using FastQC (<https://www.bioinformatics.babraham.ac.uk/projects/fastqc/>). We employed Salmon (A) to quantify transcript-level expression and EdgeR (B) to identify genes with significantly differential expression between pairs of conditions based on replicated count data from bulk RNA-seq profiling. The normalized data were applied to R package GAGE (C) for gene-set enrichment and pathway analysis. The p values were corrected for multiple testing using FDR. Heat maps were created using Morpheus from the Broad Institute (<https://software.broadinstitute.org/morpheus/>). Pathway enrichment was analyzed by ShinyGO 0.77 (<http://bioinformatics.sdstate.edu/go/>). EMT scores (e.g., MAK EMT score and PAMG classification) were computed from normalized expression matrices as previously described and used to quantitatively assess epithelial–mesenchymal states across samples.

**Statistical Analysis**

Comparisons between two experimental groups were performed using Student’s unpaired two-tailed *t* test. For multiple pairwise comparisons, unpaired *t* tests were applied with false discovery rate (FDR) correction to control for multiple testing. Categorical data, such as associations between groups or marker positivity, were analyzed using Chi-square (X^2^) tests. Overall survival differences between groups were evaluated using Kaplan-Meier survival analysis with log-rank (*Mantel-Cox*) test.

All statistical analyses and generation of quantitative graphs were performed using GraphPad Prism 10 (GraphPad Software). Data are presented as mean ± s.d., unless otherwise indicated. Statistical significance was defined as *P* < 0.05.

**References**

1.	Hingorani SR, Wang L, Multani AS, Combs C, Deramaudt TB, Hruban RH, et al. Trp53R172H and KrasG12D cooperate to promote chromosomal instability and widely metastatic pancreatic ductal adenocarcinoma in mice. Cancer Cell. 2005;7(5):469-83.

2.	Olive KP, Tuveson DA, Ruhe ZC, Yin B, Willis NA, Bronson RT, et al. Mutant p53 gain of function in two mouse models of Li-Fraumeni syndrome. Cell. 2004;119(6):847-60.

3.	Hingorani SR, Petricoin EF, Maitra A, Rajapakse V, King C, Jacobetz MA, et al. Preinvasive and invasive ductal pancreatic cancer and its early detection in the mouse. Cancer Cell. 2003;4(6):437-50.

4.	Jackson EL, Willis N, Mercer K, Bronson RT, Crowley D, Montoya R, et al. Analysis of lung tumor initiation and progression using conditional expression of oncogenic K-ras. Genes Dev. 2001;15(24):3243-8.

5.	Wilson BD, Ii M, Park KW, Suli A, Sorensen LK, Larrieu-Lahargue F, et al. Netrins promote developmental and therapeutic angiogenesis. Science. 2006;313(5787):640-4.

6.	Nabet B, Roberts JM, Buckley DL, Paulk J, Dastjerdi S, Yang A, et al. The dTAG system for immediate and target-specific protein degradation. Nat Chem Biol. 2018;14(5):431-41.
